# Supplementary material for: Analysis of mortality metrics associated with a comprehensive range of disorders in Denmark, 2000 to 2018: A population-based cohort study
Source: PLoS Med. 2022 Jun 16;19(6):e1004023. doi: 10.1371/journal.pmed.1004023 (PMC9202944; doi:10.1371/journal.pmed.1004023)
Supplement: S1 Text — (PDF) [file pmed.1004023.s001.pdf]

Analysis of mortality metrics associated with a comprehensive range of disorders in Denmark, 2000-2018: A population-based cohort study

SUPPORTING INFORMATION

S1 Text

Supplementary methods

Assessment of specific diseases based on ICD-10 classification

Specific diseases were identified through hospital contacts from the Danish National Patient Register [1,2]. The diagnostic system used during the study period was the Danish modification of the *International Classification of Diseases, Tenth Revision* (ICD-10) [3]. For this study, we considered 19 overall chapters (e.g. A00-B99: Certain infectious and parasitic diseases), 207 subchapters (e.g. A00-A09: Intestinal diseases), and 1,538 3-character categories for certain disorders (e.g. A00: Cholera), making a total list of 1,764 specific categories. Codes included in ICD-10 chapters XV (R00-R99) and XXI (Z00-Z99) were not included in this study. Specific diseases were identified as any type of diagnosis except (a) referral diagnoses (*henvisningsdiagnose*; diagnosis type 'H']) and (b) diagnoses linked to a hospital contact in which the main diagnosis is in ICD-10 chapter XXI (Z00-Z99). For each individual in the study, the date of onset for each disorder was defined as the date of first contact (inpatient, outpatient, or emergency visit).

Statistical analysis

All individuals were followed up from birth, immigration to Denmark, or January 1, 2000, whichever came last, until death, emigration from Denmark, or December 31, 2018, whichever came first. All disorders were treated as time-varying factors (i.e. individuals were considered to be exposed to a specific disorder if they were diagnosed before start of follow-up; otherwise they were considered unexposed until the first diagnosis of the disorder if any, and exposed afterwards). People with more than one disorder were considered as being exposed to each of the disorders (with different onsets depending on the date of first diagnosis for each disorder).

Number of cases and age at disease diagnosis

Number of cases was estimated as the number of individuals in the entire population with a given disorder. For disorders with at least 100 cases, the distribution of age at disorder diagnosis was estimated in percentiles, but each percentile was the average of 5 individuals to comply with data regulations. For example, if 1,000 individuals were diagnosed with a given disease, percentile 25 was an average of individuals ranked 248-252 according to age at diagnosis, while the median was an average of individuals ranked 498-502.

Number of deaths among cases and age at death

Number of deaths among cases was estimated as the number of individuals with a given diagnosis that died within the observation period. For diseases with at least 100 deaths, the distribution of age at death was estimated in percentiles, as described above.

Incidence rates

Incidence rates for each disorder were calculated as the number of individuals diagnosed with the disorder for the first time divided by the total follow-up time in person-years. In this study, incidence rates were reported per 10,000 person-years for each age group (0-5, 5-10, 10-15, …, 95-100 years).

Mortality rates and mortality rate ratios

Mortality rates for the whole population and for those diagnosed with each disorder were calculated as the number of deaths divided by the total follow-up time in person-years. Standardized mortality rates for the whole population were calculated using the distribution of sex, age (5-year categories), and calendar time (2000-2004, 2005-2009, 2010-2014, 2015-2018) of those diagnosed with each disorder. Mortality rate ratios (MRRs) with 95% confidence intervals (CIs) were estimated for external and natural causes of death and for all-causes combined, comparing persons with and without each specific disorder using Cox Proportional Hazards models, with age as the underlying time scale, and adjusting for sex and birth date (using cubic splines with 4 knots). When hazards are not proportional over time (i.e. hazard ratios depend on age), estimates from the Cox model can be interpreted as average mortality rate ratios over the entire period [4]. However, we also estimated MRRs for all causes depending on age (5-year categories) and time since diagnosis (0-6 months, 6-12 months, 1-2 years, 2-5 years, 5-10 years, 10+ years) including an interaction term with exposure in the regression models.

Life expectancy

Differences in average life expectancy between the group of persons with a specific disorder and the general population were calculated as Life Years Lost. The technical development of this method has recently been published [5,6] and a detailed account of how to implement it – with a specific R package – is available [7]. In brief, for each disorder, the expected residual lifetime was calculated at each possible age of diagnosis for the group of persons with a

previous diagnosis and for the general population of same sex and age (see Figure 1 below). The difference between these estimates was defined as differences in life expectancy at each possible age of diagnosis and was presented for all ages that include 90% of the diagnoses (from percentile 5 to percentiles 95 of the distribution of age at diagnosis). Additionally, for ages corresponding to the median and percentiles 25 and 75 of age at diagnosis, detailed figures with survival curves are presented. A weighted average of all these age-specific estimates (weighted by the number of individuals diagnosed at each age) provided a summary measure of differences in life expectancy after disorder diagnosis. Finally, these differences were divided into natural and external causes of death using a competing risks model [8]. CIs for these estimates were obtained using non-parametric bootstrap with 500 iterations. Differences in life expectancy and life years lost are presented for diseases with at least 100 individuals diagnosed and at least 20 deaths, as long as there were enough diagnosed individuals at older ages of follow-up (the survival probability must have been lower than 10% when there were less than 10 diagnosed individuals at risk). The main reason to compare those with a given disease to the general population – and not to persons without the disease – is that the number of Life Years Lost at a given age, e.g. 45 years, is estimated using mortality rates at ages 45 years and beyond. By choosing persons without the disease as a comparison group, we would assume that someone who has not experienced the disease at age 45, would remain free of the disease until death. Although it might seem problematic to include persons with a disease in both the diseased and reference groups, this is analogous to widely used (and classic) standardized mortality ratios (SMRs), which compare mortality in a group of persons to the one in the general population. In any case, differences in life expectancy would be even larger if the comparison group were persons without the disease.

Effect of air pollution on observed mortality differences

The study population was linked with information on residential exposure to air pollutants during the year before start of follow-up [9]. Consequently, follow-up for these analyses started on the date of the first birthday, one year after immigration to Denmark, or January 1, 2000, whichever came last. Individuals who were born in or immigrated to Denmark after December 31, 2017 (118,553; 1.6%), or who died (3,717; <0.1%) or emigrated (266,197; 3.6%) during the first year of follow-up were excluded from the analyses, leaving a sample of 6,990,131 individuals. The daily mean exposure levels of nitrogen dioxide (NO_2) and atmospheric particulate matter with a diameter of less than 2.5 micrometers ($PM_{2.5}$) at each

individual's home address (based on modelling at 1 km x 1 km resolution) during the year before follow-up were available for 6,984,647 individuals (99.9%). Models estimating MRRs for all-cause mortality were replicated in this study population with and without adjustment for mean NO_2 and $PM_{2.5}$, included in the models as continuous z-scores.

Figure 1. Worked example of the Life Years Lost method.

Person A is diagnosed with a specific disorder at age 30 years. Based on mortality rates for individuals with that disorder, the remaining expected life expectancy at age 30 years is estimated. Additionally, remaining expected life expectancy at age 30 years is estimated also for the general population of same sex (B). The difference between the two estimates is the life years lost at age 30 years. An analogous example for life years lost at age 17 years is provided for person C and matched population D. The overall estimate of life years lost is based on the average of all individual estimates, i.e. a weighted average of age-specific estimates weighted by the number of individuals diagnosed at each age.

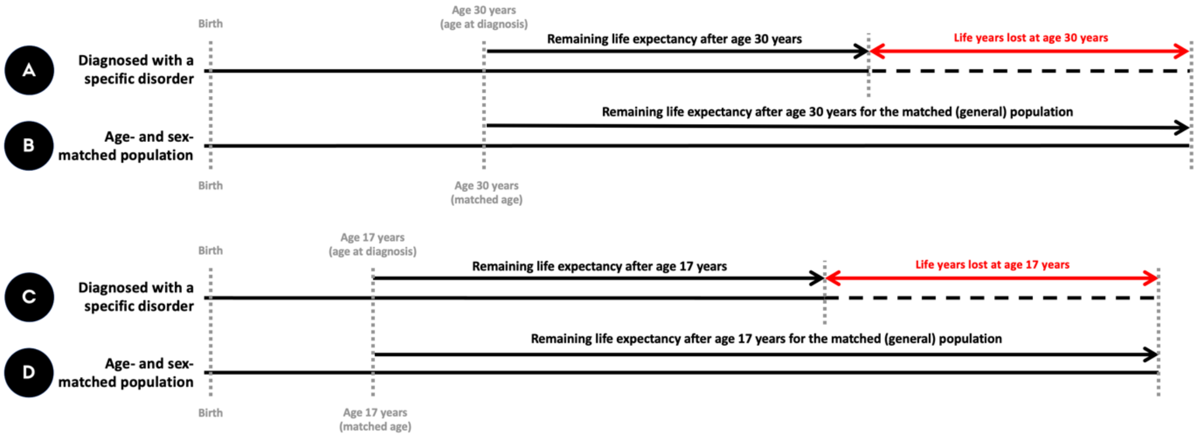

REFERENCES

1. Lynge E, Sandegaard JL, Rebolj M. The Danish National Patient Register. Scandinavian Journal of Public Health. 2011;39: 30–33. doi:10.1177/1403494811401482

2. Mors O, Perto GP, Mortensen PB. The Danish Psychiatric Central Research Register. Scand J Public Health. 2011;39: 54–57. doi:10.1177/1403494810395825

3. Sundhedsdatastyrelsen. Klassifikation af sygdomme. [cited 27 Oct 2020]. Available: https://sundhedsdatastyrelsen.dk/da/rammer-og-retningslinjer/om-klassifikationer/sks-klassifikationer/klassifikation-sygdomme

4. Xu R, O'Quigley J. Estimating average regression effect under non-proportional hazards. Biostatistics. 2000;1: 423–439. doi:10.1093/biostatistics/1.4.423

5. Andersen PK. Life years lost among patients with a given disease. Stat Med. 2017;36: 3573–3582. doi:10.1002/sim.7357

6. Erlangsen A, Andersen PK, Toender A, Laursen TM, Nordentoft M, Canudas-Romo V. Cause-specific life-years lost in people with mental disorders: a nationwide, register-based cohort study. Lancet Psychiatry. 2017;4: 937–945. doi:10.1016/s2215-0366(17)30429-7

7. Plana-Ripoll O, Canudas-Romo V, Weye N, Laursen TM, McGrath JJ, Andersen PK. lillies: an R package for the estimation of excess Life Years Lost among patients with a given disease or condition. Emilsson L, editor. Plos One. 2020;15: e0228073. doi:10.1371/journal.pone.0228073

8. Andersen PK. Decomposition of number of life years lost according to causes of death. Stat Med. 2013;32: 5278–5285. doi:10.1002/sim.5903

9. Raaschou-Nielsen O, Thorsteinson E, Antonsen S, Holst GJ, Sigsgaard T, Geels C, et al. Long-term exposure to air pollution and mortality in the Danish population a nationwide study. EClinicalMedicine. 2020;28: 100605. doi:10.1016/j.eclinm.2020.100605
