# Supplementary material for: Analysis of mortality metrics associated with a comprehensive range of disorders in Denmark, 2000 to 2018: A population-based cohort study
Source: PLoS Med. 2022 Jun 16;19(6):e1004023. doi: 10.1371/journal.pmed.1004023 (PMC9202944; doi:10.1371/journal.pmed.1004023)
Supplement: S2 Text — (PDF) [file pmed.1004023.s002.pdf]

Analysis of mortality metrics associated with a comprehensive range of disorders in Denmark, 2000-2018: A population-based cohort study

SUPPORTING INFORMATION

S2 Text

Pre-specified analysis plan

This document was posted on Open Science Framework (https://osf.io/zafhu) on November 2nd, 2020 before having access to the data.

Plana-Ripoll O, Dreier JW, Pedersen CB, Pedersen MG, Agerbo E, Mortensen PB, et al. The Danish Atlas of Disease Mortality. OSF. 2021. doi:10.17605/OSF.IO/ZAFHU

The Danish Atlas of Disease Mortality
ANALYSIS PLAN

Oleguer Plana-Ripoll, Julie Werenberg Dreier, Carsten Bøcker Pedersen, Marianne Giørtz Pedersen, Esben Agerbo, Preben Bo Mortensen, John J. McGrath

Introduction

The main aim of the Danish Atlas of Disease Mortality is to estimate mortality-related metrics for specific diseases based on hospital contacts between 1995 and 2018 in Denmark. The atlas will provide a comprehensive database that can inform future research, and guide decisions about health resource allocations. Based on related projects[1] and other prior studies,[2–4] we expect to see wide variation in mortality-related metrics between types of disorders and (to a lesser extent) between sexes.

Planned secondary analyses

Additionally, we will explore what proportion of the observed differences in mortality between those with and without a specific disease could be explained by measures of air pollution. We predict that adjustment for cumulative exposure to higher levels of air pollution will be associated with smaller differences in mortality.

Study population and follow-up

We will design a population-based cohort study including all persons living in Denmark at some point between January 1, 2000 and December 31, 2018 (i.e. we will include all persons living in Denmark on January 1, 2000 and those born or immigrating after that date). Since 1968, the Danish Civil Registration System[5] has maintained information on all residents, including sex, date of birth, continuously updated information on vital status, and a unique personal identification number that can be used to link information from various national registries.

Assessment of specific diseases based on ICD-10 classification

Specific diseases will be identified through hospital contacts on or after January 1, 1995. This will allow a period of at least 5 years to identify individuals with diseases diagnosed before the start of the follow-up. This information will be obtained from the Danish National Patient Register,[6] which contains data on all admissions to hospital in- and outpatient facilities, as well as emergency departments, since 1995 (including contacts from psychiatric departments, available through the Danish Psychiatric Central Research Register[7]). The diagnostic system used during this period is the Danish modification of the *International Classification of Diseases, Tenth Revision* (ICD-10).[8] All diseases will be identified using 19 chapters (e.g. A00-B99: Certain infectious and parasitic diseases), 207 subchapters (e.g. A00-A09: Intestinal diseases), and 1,538 3-level codes for certain diseases (e.g. A00: Cholera), making a total list of 1,764 specific disease categories

for which mortality metrics will be estimated (see complete list in supplementary file "List_of_diseases.xlsx"). Codes included in ICD-10 chapters XV (R00-R99) and XXI (Z00-Z99) will not be included in this study. Specific diseases will be identified as any type of diagnosis except (a) referral diagnoses (*henvisningsdiagnose*; diagnosis type 'H']) and (b) diagnoses linked to a hospital contact in which the main diagnosis is in ICD-10 chapter XXI (Z00-Z99). In addition to the 1,764 diseases based on ICD-10, we will consider additional diseases based on combinations of several ICD-10 codes in order to facilitate comparisons with previous publications from our group[9] (see complete list in supplementary file "Additional_disorders.xlsx"). For each individual in the study, the date of onset for each disease will be defined as the date of first contact (inpatient, outpatient, or emergency visit).

Mortality

Information on date and primary cause of death will be obtained from the Danish Registry of Causes of Death.[10] All deaths will be categorized in two non-overlapping groups according to ICD-10 codes: external causes of death, which include suicide (X60-X84, Y87.0), homicide (X85-Y09, Y87.1), and accidents (V01-X59, Y10-Y86, Y87.2, Y88-Y89); and natural causes of death, which include all other causes.

Statistical analysis

All individuals will be followed up from birth, immigration, or January 1, 2000, whichever comes last, until death, emigration from Denmark, or December 31, 2018, whichever comes first. All diseases will be treated as time-varying factors (i.e. individuals will be considered to be exposed to a specific disease if they are diagnosed before start of follow-up; otherwise they will be considered unexposed until the first diagnosis of the disease, and exposed afterwards). People with more than one disease will be considered as being exposed to each of the diseases (with different onsets depending on the date of first diagnosis for each disease). All mortality metrics will be estimated for males and females combined, and stratified on sex.

Number of cases and age at disease diagnosis

Number of cases will be estimated as the number of individuals in the entire population with a given disease. For diseases with at least 100 cases, the distribution of age at disease diagnosis will be estimated in percentiles, but each percentile will be the average of 5 individuals to comply with data regulations. For example, if 1,000 individuals are diagnosed with a given disease, percentile 25 will be an average of individuals ranked 248-252 according to age at diagnosis, while the median will be an average of individuals ranked 498-502.

Number of deaths among cases and age at death

Number of deaths among cases will be estimated as the number of individuals with a given diagnosis that die within the observation period. For diseases with at least 100 deaths, the distribution of age at death will be estimated in percentiles, as described above.

Mortality rates and mortality rate ratios

Mortality rates for the whole population and for those diagnosed with each disease will be calculated as the number of deaths divided by the total follow-up time in person-years. Standardized mortality rates for the whole population will be calculated using the distribution of sex, age (5-year categories), and calendar time (2000-2004, 2005-2009, 2010-2014, 2015-2018) of those diagnosed with each disease. Mortality rate ratios (MRRs) with 95% confidence intervals (CIs) will be estimated for each specific cause of death and for all-causes combined, comparing persons with and without each specific disease using Cox Proportional Hazards models, with age as the underlying time scale, and adjusting for sex and birth date (using cubic splines with 4 knots). Calendar year is also controlled for in these models given the combination of age as time scale and adjustment for birth date. Mortality rates and MRRs will be presented for diseases with at least 100 individuals diagnosed and at least 20 deaths.

Life expectancy

Differences in average life expectancy between the group of persons with a specific disease and the general population will be calculated as Life Years Lost. The technical development of this method has recently been published[11,12] and a detailed account of how to implement it – with a specific R package – is available.[13] In brief, for each disease, the expected residual lifetime will be calculated at each possible age of diagnosis for the group of persons with a previous diagnosis and for the general population of same sex and age. The difference between these estimates will be defined as differences in life expectancy at each possible age of diagnosis and will be presented for all ages that include 90% of the diagnoses (from percentile 5 to percentiles 95 of the distribution of age at diagnosis). Additionally, for ages corresponding to the median and percentiles 25 and 75 of age at diagnosis, detailed figures with survival curves will be presented. A weighted average of all these age-specific estimates (weighted by the number of diagnosed at each age) will provide a summary measure of differences in life expectancy after disease diagnosis. Finally, these differences will be decomposed into natural and external causes of death using a competing risks model.[14] CIs for these estimates will be obtained using non-parametric bootstrap with 1,000 iterations. Differences in life expectancy and life years lost will be presented for diseases with at least 100 individuals diagnosed and at least 20 deaths, as long as there are enough diagnosed individuals at older ages of follow-up (the survival probability must be lower than 10% when there are less than 10 diagnosed individuals at risk).

Effect of air pollution on observed mortality differences

In order to estimate the potential effect of air pollution on MRRs, additional sensitivity analyses will be carried out. The study population will be linked with information on residential exposure to air pollutants during the year before start of follow-up. Consequently, follow-up for these analyses will start on date of first birthday, one year after immigration to Denmark, or January 1, 2000, whichever comes last (individuals dying during the first year of life or in the first year after immigrating to Denmark will be excluded from the analyses). The daily mean exposure levels of nitrogen dioxide (NO2) and atmospheric particulate matter that have a diameter of less than 2.5 micrometers (PM2.5) at each individual's home address (1 km x 1 km

resolution) during the year before follow-up will be summed and subsequently divided by the number of measurements available. Models estimating MRRs for all-cause mortality will be replicated in this study population with and without adjustment for mean NO2 and PM2.5, included in the models as continuous z-scores.

All analyses will be performed using R version 4.0.3 or higher.

Ethics and data protection

All analyses will be performed on the secured platform of the Danish Health Data Authority (*Sundhedsdatastyrelsen*). The Danish Data Protection Agency and the Danish Health Data Authority approved this study. According to Danish law, informed consent is not required for register-based studies. All data will be de-identified and not recognizable at an individual level.

Code and data availability

All programming code and summary outcomes will be publicly available for downloading. Additionally, we will develop an interactive webpage to visualize all results from this study. Due to data protection laws, the underlying person-level data will not be available to other researchers.

Funding

The project is supported by the Danish National Research Foundation (Niels Bohr Professorship to John McGrath). Oleguer Plana-Ripoll has received funding from the European Union's Horizon 2020 research and innovation programme under the Marie Sklodowska-Curie grant agreement No 837180.

Final remarks

This analysis plan will be posted on Open Science Framework (https://osf.io/) and logged with a date stamp. Note that project participants can make decisions to amend or change this analysis plan (any updates will be posted and date stamped on Open Science Framework).

References

1. Vos T, Lim SS, Abbafati C, et al. Global burden of 369 diseases and injuries in 204 countries and territories, 1990–2019: a systematic analysis for the Global Burden of Disease Study 2019. *Lancet*. 2020;396(10258):1204-1222. doi:10.1016/S0140-6736(20)30925-9
2. Knudsen AK, Allebeck P, Tollånes MC, et al. Life expectancy and disease burden in the Nordic countries: results from the Global Burden of Diseases, Injuries, and Risk Factors Study 2017. *Lancet Public Heal*. November 2019. doi:10.1016/S2468-2667(19)30224-5
3. Plana-Ripoll O, Pedersen CB, Agerbo E, et al. A comprehensive analysis of mortality-related health metrics associated with mental disorders: a nationwide, register-based cohort study. *Lancet*. 2019;394(10211):1827-1835. doi:10.1016/S0140-6736(19)32316-5

4. Weye N, Momen NC, Christensen MK, et al. Association of Specific Mental Disorders With Premature Mortality in the Danish Population Using Alternative Measurement Methods. *JAMA Netw open*. 2020;3(6):e206646. doi:10.1001/jamanetworkopen.2020.6646
5. Pedersen CB. The Danish Civil Registration System. *Scand J Public Health*. 2011;39(7 Suppl):22-25. doi:10.1177/1403494810387965
6. Lynge E, Sandegaard JL, Rebolj M. The Danish National Patient Register. *Scand J Public Health*. 2011;39(7 Suppl):30-33. doi:10.1177/1403494811401482
7. Mors O, Perto GP, Mortensen PB. The Danish Psychiatric Central Research Register. *Scand J Public Heal*. 2011;39(7 Suppl):54-57. doi:10.1177/1403494810395825
8. Sundhedsdatastyrelsen. Klassifikation af sygdomme. https://sundhedsdatastyrelsen.dk/da/rammer-og-retningslinjer/om-klassifikationer/sks-klassifikationer/klassifikation-sygdomme. Accessed October 27, 2020.
9. Momen NC, Plana-Ripoll O, Agerbo E, et al. Association between Mental Disorders and Subsequent Medical Conditions. *N Engl J Med*. 2020;382(18):1721-1731. doi:10.1056/NEJMoa1915784
10. Helweg-Larsen K. The Danish Register of Causes of Death. *Scand J Public Health*. 2011;39(7 Suppl):26-29. doi:10.1177/1403494811399958
11. Andersen PK. Life years lost among patients with a given disease. *Stat Med*. 2017;36(22):3573-3582. doi:10.1002/sim.7357
12. Erlangsen A, Andersen PK, Toender A, Laursen TM, Nordentoft M, Canudas-Romo V. Cause-specific life-years lost in people with mental disorders: a nationwide, register-based cohort study. *Lancet Psychiatry*. 2017;4(12):937-945. doi:10.1016/s2215-0366(17)30429-7
13. Plana-Ripoll O, Canudas-Romo V, Weye N, Laursen TM, McGrath JJ, Andersen PK. lillies: an R package for the estimation of excess Life Years Lost among patients with a given disease or condition. Emilsson L, ed. *PLoS One*. 2020;15(3):e0228073. doi:10.1371/journal.pone.0228073
14. Andersen PK. Decomposition of number of life years lost according to causes of death. *Stat Med*. 2013;32(30):5278-5285. doi:10.1002/sim.5903
