# Supplementary material for: Analysis of mortality metrics associated with a comprehensive range of disorders in Denmark, 2000 to 2018: A population-based cohort study
Source: PLoS Med. 2022 Jun 16;19(6):e1004023. doi: 10.1371/journal.pmed.1004023 (PMC9202944; doi:10.1371/journal.pmed.1004023)
Supplement: S3 Table — (PDF) [file pmed.1004023.s005.pdf]

S3 Table. List of 39 selected conditions covering 10 broad categories of disorders.

Category	Disorder	ICD-10 codes
CIRCULATORY SYSTEM		
	Hypertension	I10-I13, I15
	Dyslipidemia	E78
	Ischemic heart disease	I20-I25
	Atrial fibrillation	I48
	Heart failure	I50
	Peripheral artery occlusive disease	I70-I74
	Stroke	I60-I64, I69
ENDOCRINE SYSTEM		
	Diabetes mellitus	E10-E14
	Thyroid disorder	E00-E05, E06.1-E06.9, E07
	Gout	E79, M10
PULMONARY SYSTEM AND ALLERGY		
	Chronic pulmonary disease	J40-J47
	Allergy	J30.1-J30.4, L23, L50.0, T78.0, T78.2, T78.4
GASTROINTESTINAL SYSTEM		
	Ulcer/chronic gastritis	K22.1, K25-K28, K29.3-K29.5
	Chronic liver disease	B16-B19, K70, K74, K76.6, I85
	Inflammatory bowel disease	K50-K51
	Diverticular disease of intestine	K57
UROGENITAL SYSTEM		
	Chronic kidney disease	N03, N11, N18-N19
	Prostate disorders	N40
MUSCULOSKELETAL SYSTEM		
	Connective tissue disorders	M05-M06, M08-M09, M30-M36, D86
	Osteoporosis	M80-M82
HEMATOLOGICAL SYSTEM		
	HIV/AIDS	B20-B24
	Anemias	D50-D53, D55-D59, D60-D61, D63-D64
CANCERS		
	Cancers	C00-C43, C45-C97
NEUROLOGICAL SYSTEM		
	Vision problem	H40, H25, H54
	Hearing problem	H90-H91, H93.1
	Migraine	G43
	Epilepsy	G40-G41
	Parkinson's disease	G20-G22
	Multiple sclerosis	G35
	Neuropathies	G50-G64
MENTAL DISORDERS		
	Mental disorders	F00-F99
