# Supplementary material for: Analysis of mortality metrics associated with a comprehensive range of disorders in Denmark, 2000 to 2018: A population-based cohort study
Source: PLoS Med. 2022 Jun 16;19(6):e1004023. doi: 10.1371/journal.pmed.1004023 (PMC9202944; doi:10.1371/journal.pmed.1004023)
Supplement: S4 Table — LYLs, life years lost; MRR, mortality rate ratio. (PDF) [file pmed.1004023.s006.pdf]

Analysis of mortality metrics associated with a comprehensive range of disorders in Denmark, 2000-2018: A population-based cohort study (Supporting information – S4 Table)

S4 Table. For 39 selected conditions covering 10 broad categories: Number of diagnosed, age at diagnosis (median and interquartile range), number of deaths among the diagnosed, age at death (median and interquartile range), Mortality Rate Ratios (MRR; with 95% confidence interval) and Life Years Lost (LYL; with 95% confidence interval) for all causes of death. Estimates are not shown if they are based on less than 100 individuals diagnosed or less than 20 deaths; for Life Years Lost, estimates are not shown if there were not enough individuals at older ages of follow-up.

Disorder	N	Age at diagnosis	Deaths	Age at death	MRR	LYL
Circulatory system	1,431,041	66.6 (55.5-76.6)	629,860	81.6 (73.3-88.0)	2.9 (2.9-2.9)	3.8 (3.8-3.8)
Hypertension	777,878	67.5 (57.0-76.9)	298,758	81.6 (73.4-88.0)	1.8 (1.7-1.8)	2.9 (2.8-2.9)
Dislipidemia	280,111	64.7 (55.4-73.3)	84,211	78.4 (70.8-84.9)	1.4 (1.4-1.4)	2.1 (2.1-2.2)
Ischemic heart disease	503,434	67.8 (57.4-77.3)	250,857	81.7 (74.1-87.8)	1.9 (1.9-1.9)	3.7 (3.6-3.7)
Atrial fibrillation	362,525	74.5 (65.3-82.3)	205,105	83.7 (76.8-89.3)	2.3 (2.2-2.3)	4.2 (4.1-4.2)
Heart failure	251,887	76.2 (66.9-83.7)	183,015	82.8 (75.5-88.7)	3.3 (3.3-3.3)	6.5 (6.4-6.5)
Peripheral artery occlusive disease	225,408	70.3 (61.0-78.2)	127,307	79.9 (72.7-86.1)	2.6 (2.6-2.6)	6.1 (6.0-6.1)
Stroke	358,431	72.4 (61.2-81.1)	217,881	82.0 (74.1-88.0)	2.8 (2.8-2.8)	6.2 (6.2-6.2)
Endocrine system	605,203	60.5 (46.9-72.6)	211,165	79.6 (70.8-86.5)	2.0 (2.0-2.0)	4.7 (4.6-4.7)
Diabetes Mellitus	332,189	63.5 (51.7-73.9)	145,684	78.2 (69.6-85.2)	2.3 (2.3-2.3)	6.1 (6.1-6.2)
Thyroid disorder	265,551	55.8 (41.8-70.5)	64,201	82.2 (74.1-88.3)	1.5 (1.4-1.5)	2.4 (2.4-2.5)
Gout	54,039	67.2 (54.0-77.9)	22,715	81.0 (72.7-87.5)	2.0 (2.0-2.0)	4.7 (4.6-4.8)
Pulmonary system and allergy	690,345	50.7 (19.6-69.4)	202,644	78.6 (70.9-84.9)	2.7 (2.7-2.8)	7.5 (7.5-7.6)
Chronic pulmonary disease	568,030	55.7 (18.7-71.7)	194,280	78.7 (71.2-84.9)	2.9 (2.9-3.0)	8.3 (8.2-8.3)
Allergy	170,739	34.0 (18.1-50.7)	12,400	74.5 (63.7-84.0)	1.1 (1.1-1.1)	0.3 (0.1-0.5)
Gastrointestinal system	474,333	63.0 (49.0-74.6)	186,028	79.5 (68.9-87.0)	2.0 (2.0-2.1)	6.0 (6.0-6.1)
Ulcer/chronic gastritis	194,212	67.3 (53.3-78.3)	104,338	80.7 (71.5-87.5)	2.2 (2.2-2.3)	6.6 (6.5-6.6)
Chronic liver disease	70,295	53.8 (42.8-63.6)	35,935	63.1 (55.0-71.5)	7.8 (7.8-7.9)	17.1 (17.0-17.1)
Inflamatory bowel disease	75,207	43.3 (28.3-60.5)	13,199	78.0 (68.1-85.0)	1.5 (1.4-1.5)	3.2 (3.1-3.4)
Diverticular disease of intestine	180,017	68.5 (58.7-76.9)	56,676	83.5 (76.5-89.1)	1.2 (1.2-1.2)	1.3 (1.2-1.3)
Urogenital system	255,126	71.1 (61.9-79.0)	139,466	81.7 (74.5-87.4)	1.9 (1.8-1.9)	4.5 (4.4-4.5)
Chronic kidney disease	119,289	72.8 (59.3-81.6)	71,125	80.2 (71.6-86.6)	4.2 (4.1-4.2)	9.5 (9.5-9.6)
Prostate disorders	150,437	70.9 (63.5-77.9)	79,099	83.0 (77.1-88.1)	1.2 (1.2-1.2)	0.8 (0.7-0.8)
Musculoskeletal system	395,014	67.0 (53.9-77.0)	148,073	81.7 (73.3-88.1)	1.9 (1.8-1.9)	4.4 (4.3-4.4)
Connective tissue disorders	177,308	58.7 (39.5-72.2)	51,389	81.2 (73.2-87.3)	1.5 (1.5-1.5)	3.3 (3.2-3.4)
Osteoporosis	246,366	71.4 (62.0-79.9)	109,978	82.1 (73.7-88.5)	2.0 (2.0-2.0)	4.5 (4.4-4.5)
Hematological system	282,890	72.8 (56.8-82.4)	174,735	81.4 (71.9-88.2)	3.8 (3.8-3.8)	9.7 (9.6-9.7)
HIV/AIDS	6,531	37.9 (30.9-46.9)	1,222	53.5 (44.8-63.9)	3.7 (3.5-3.9)	-
Anemias	276,971	73.2 (58.3-82.5)	173,777	81.5 (72.1-88.2)	3.8 (3.8-3.8)	9.6 (9.5-9.6)
Cancers	640,038	67.7 (57.7-76.2)	364,443	75.5 (66.5-83.3)	4.9 (4.9-5.0)	9.9 (9.9-9.9)
Neurological system	1,283,880	64.8 (47.8-75.6)	442,749	83.7 (75.7-89.7)	1.2 (1.2-1.2)	1.7 (1.7-1.8)
Vision problem	511,549	74.5 (67.2-80.6)	235,433	85.5 (79.3-90.6)	1.1 (1.1-1.1)	0.8 (0.8-0.9)
Hearing problem	477,430	69.3 (56.3-78.3)	202,206	86.3 (79.7-91.4)	0.9 (0.9-0.9)	-0.1 (-0.1--0.1)
Migraine	81,142	36.4 (22.9-48.6)	4,077	70.5 (58.4-81.7)	1.0 (1.0-1.0)	-0.6 (-0.9--0.4)
Epilepsy	118,527	45.2 (19.4-65.7)	40,944	72.5 (60.3-82.2)	3.4 (3.3-3.4)	13.5 (13.4-13.6)
Parkinson's disease	31,465	74.9 (67.6-80.9)	20,844	81.8 (76.4-86.6)	2.7 (2.7-2.7)	5.5 (5.4-5.5)
Multiple sclerosis	20,917	44.3 (34.4-53.9)	4,612	66.8 (57.9-75.4)	2.8 (2.7-2.9)	8.8 (8.5-9.0)
Neuropathies	317,936	53.8 (41.6-66.5)	60,165	76.8 (66.3-85.6)	1.3 (1.3-1.3)	2.0 (1.9-2.0)
Mental disorders	1,128,977	41.4 (21.6-65.6)	318,197	80.2 (67.5-87.7)	3.4 (3.3-3.4)	9.5 (9.5-9.5)
