# Supplementary material for: Analysis of mortality metrics associated with a comprehensive range of disorders in Denmark, 2000 to 2018: A population-based cohort study
Source: PLoS Med. 2022 Jun 16;19(6):e1004023. doi: 10.1371/journal.pmed.1004023 (PMC9202944; doi:10.1371/journal.pmed.1004023)
Supplement: S5 Table — All estimates are adjusted for age, sex, and birth date. MRR, mortality rate ratio. (PDF) [file pmed.1004023.s007.pdf]

Analysis of mortality metrics associated with a comprehensive range of disorders in Denmark, 2000-2018: A population-based cohort study (Supporting information – S5 Table)

S5 Table. Mortality rate ratios for all causes of death for 39 selected conditions covering 10 broad categories with and without adjustment for air pollution during the year before start of follow-up. All estimates are adjusted for age, sex and birth date.

Disorder	Mortality Rate Ratios	
	Not adjusted	Adjusted
Circulatory system	2.9 (2.9-2.9)	2.9 (2.9-2.9)
Hypertension	1.8 (1.7-1.8)	1.8 (1.7-1.8)
Dislipidemia	1.4 (1.4-1.4)	1.4 (1.4-1.4)
Ischemic heart disease	1.9 (1.9-1.9)	1.9 (1.9-1.9)
Atrial fibrillation	2.3 (2.2-2.3)	2.3 (2.2-2.3)
Heart failure	3.3 (3.3-3.3)	3.3 (3.3-3.3)
Peripheral artery occlusive disease	2.6 (2.6-2.6)	2.6 (2.6-2.6)
Stroke	2.8 (2.8-2.8)	2.8 (2.8-2.8)
Endocrine system	2.0 (2.0-2.0)	2.0 (2.0-2.0)
Diabetes Mellitus	2.3 (2.3-2.3)	2.3 (2.3-2.3)
Thyroid disorder	1.5 (1.4-1.5)	1.5 (1.4-1.5)
Gout	2.0 (2.0-2.0)	2.0 (2.0-2.0)
Pulmonary system and allergy	2.7 (2.7-2.8)	2.7 (2.7-2.7)
Chronic pulmonary disease	2.9 (2.9-3.0)	2.9 (2.9-2.9)
Allergy	1.1 (1.1-1.1)	1.1 (1.1-1.1)
Gastrointestinal system	2.0 (2.0-2.1)	2.0 (2.0-2.1)
Ulcer/chronic gastritis	2.2 (2.2-2.3)	2.2 (2.2-2.3)
Chronic liver disease	7.8 (7.8-7.9)	7.8 (7.7-7.8)
Inflamatory bowel disease	1.5 (1.4-1.5)	1.5 (1.4-1.5)
Diverticular disease of intestine	1.2 (1.2-1.2)	1.2 (1.2-1.2)
Urogenital system	1.9 (1.8-1.9)	1.9 (1.8-1.9)
Chronic kidney disease	4.2 (4.1-4.2)	4.1 (4.1-4.2)
Prostate disorders	1.2 (1.2-1.2)	1.2 (1.2-1.2)
Musculoskeletal system	1.9 (1.8-1.9)	1.8 (1.8-1.9)
Connective tissue disorders	1.5 (1.5-1.5)	1.5 (1.5-1.5)
Osteoporosis	2.0 (2.0-2.0)	2.0 (2.0-2.0)
Hematological system	3.8 (3.8-3.8)	3.8 (3.8-3.8)
HIV/AIDS	3.7 (3.5-3.9)	3.5 (3.3-3.7)
Anemias	3.8 (3.8-3.8)	3.8 (3.8-3.8)
Cancers	4.9 (4.9-4.9)	4.9 (4.9-4.9)
Neurological system	1.2 (1.2-1.2)	1.2 (1.2-1.2)
Vision problem	1.1 (1.1-1.1)	1.1 (1.1-1.1)
Hearing problem	0.9 (0.9-0.9)	0.9 (0.9-0.9)
Migraine	1.0 (1.0-1.0)	1.0 (1.0-1.0)
Epilepsy	3.4 (3.3-3.4)	3.3 (3.3-3.4)
Parkinson's disease	2.7 (2.7-2.7)	2.7 (2.7-2.7)
Multiple sclerosis	2.8 (2.7-2.9)	2.8 (2.7-2.9)
Neuropathies	1.3 (1.3-1.3)	1.3 (1.3-1.3)
Mental disorders	3.4 (3.3-3.4)	3.4 (3.3-3.4)
