# Supplementary material for: Analysis of mortality metrics associated with a comprehensive range of disorders in Denmark, 2000 to 2018: A population-based cohort study
Source: PLoS Med. 2022 Jun 16;19(6):e1004023. doi: 10.1371/journal.pmed.1004023 (PMC9202944; doi:10.1371/journal.pmed.1004023)
Supplement: S6 Table — Estimates are not shown if they are based on less than 100 individuals diagnosed or less than 20 deaths; for LYLs, estimates are not shown if there were not enough individuals at older ages of follow-up. LYLs, life years lost; MRR, mortality rate ratio. (PDF) [file pmed.1004023.s008.pdf]

Analysis of mortality metrics associated with a comprehensive range of disorders in Denmark, 2000-2018: A population-based cohort study (Supporting information – S6 Table)

S6 Table. Mortality rate ratios and Life Years Lost for natural and external causes of death for 39 selected conditions covering 10 broad categories. Estimates are not shown if they are based on less than 100 individuals diagnosed or less than 20 deaths; for Life Years Lost, estimates are not shown if there were not enough individuals at older ages of follow-up.

Disorder	Mortality Rate Ratios		Life Years Lost	
	**Natural causes**	**External causes**	**Natural causes**	**External causes**
Circulatory system	2.9 (2.9-3.0)	2.0 (2.0-2.1)	3.8 (3.8-3.8)	0.0 (0.0-0.0)
Hypertension	1.8 (1.8-1.8)	1.5 (1.5-1.5)	2.9 (2.9-2.9)	0.0 (-0.1-0.0)
Dislipidemia	1.4 (1.4-1.5)	1.2 (1.1-1.2)	2.2 (2.2-2.3)	-0.1 (-0.1--0.1)
Ischemic heart disease	1.9 (1.9-1.9)	1.4 (1.4-1.5)	3.7 (3.7-3.8)	-0.1 (-0.1--0.1)
Atrial fibrillation	2.3 (2.3-2.3)	1.8 (1.7-1.9)	4.2 (4.2-4.3)	-0.1 (-0.1-0.0)
Heart failure	3.3 (3.3-3.3)	2.2 (2.1-2.3)	6.6 (6.5-6.6)	-0.1 (-0.1--0.1)
Peripheral artery occlusive disease	2.6 (2.6-2.6)	1.7 (1.6-1.8)	6.2 (6.1-6.2)	-0.1 (-0.1--0.1)
Stroke	2.8 (2.8-2.8)	2.3 (2.3-2.4)	6.1 (6.0-6.1)	0.1 (0.1-0.1)
Endocrine system	2.1 (2.0-2.1)	1.6 (1.5-1.6)	4.7 (4.7-4.8)	0.0 (-0.1-0.0)
Diabetes Mellitus	2.3 (2.3-2.4)	1.7 (1.7-1.8)	6.1 (6.1-6.2)	0.0 (0.0-0.0)
Thyroid disorder	1.5 (1.4-1.5)	1.3 (1.2-1.3)	2.5 (2.4-2.6)	-0.1 (-0.1-0.0)
Gout	2.0 (2.0-2.0)	1.5 (1.4-1.7)	4.8 (4.6-4.9)	0.0 (-0.1-0.0)
Pulmonary system and allergy	2.8 (2.8-2.8)	1.9 (1.9-2.0)	7.4 (7.3-7.4)	0.1 (0.1-0.2)
Chronic pulmonary disease	3.0 (3.0-3.0)	2.0 (2.0-2.1)	8.1 (8.0-8.1)	0.2 (0.2-0.3)
Allergy	1.1 (1.1-1.1)	1.2 (1.1-1.2)	0.3 (0.1-0.4)	0.1 (-0.1-0.2)
Gastrointestinal system	2.0 (2.0-2.0)	2.2 (2.2-2.3)	5.6 (5.6-5.7)	0.4 (0.4-0.4)
Ulcer/chronic gastritis	2.2 (2.2-2.3)	2.1 (2.0-2.2)	6.3 (6.3-6.4)	0.2 (0.2-0.3)
Chronic liver disease	7.8 (7.7-7.9)	9.3 (8.9-9.8)	15.2 (15.0-15.3)	1.9 (1.7-2.0)
Inflamatory bowel disease	1.5 (1.4-1.5)	1.2 (1.1-1.3)	3.3 (3.1-3.5)	-0.1 (-0.2-0.0)
Diverticular disease of intestine	1.2 (1.2-1.2)	1.2 (1.2-1.3)	1.3 (1.2-1.3)	0.0 (0.0-0.0)
Urogenital system	1.9 (1.9-1.9)	1.6 (1.5-1.6)	4.2 (4.2-4.3)	0.3 (0.2-0.3)
Chronic kidney disease	4.2 (4.1-4.2)	3.8 (3.6-4.0)	8.9 (8.8-9.0)	0.6 (0.5-0.7)
Prostate disorders	1.2 (1.2-1.2)	1.1 (1.1-1.2)	0.8 (0.7-0.8)	0.0 (-0.1-0.0)
Musculoskeletal system	1.9 (1.8-1.9)	1.8 (1.7-1.8)	4.4 (4.3-4.4)	0.0 (0.0-0.0)
Connective tissue disorders	1.5 (1.5-1.5)	1.3 (1.3-1.4)	3.3 (3.2-3.4)	0.0 (-0.1-0.0)
Osteoporosis	2.0 (2.0-2.0)	2.1 (2.0-2.1)	4.5 (4.4-4.5)	0.0 (0.0-0.0)
Hematological system	3.9 (3.8-3.9)	2.5 (2.5-2.6)	9.7 (9.6-9.7)	0.0 (0.0-0.1)
HIV/AIDS	3.6 (3.4-3.8)	5.2 (4.4-6.1)	-	-
Anemias	3.9 (3.8-3.9)	2.5 (2.4-2.6)	9.6 (9.5-9.6)	0.0 (0.0-0.0)
Cancers	5.1 (5.1-5.1)	1.4 (1.3-1.4)	10.3 (10.3-10.4)	-0.4 (-0.4--0.4)
Neurological system	1.2 (1.2-1.2)	1.3 (1.3-1.4)	1.6 (1.6-1.6)	0.1 (0.1-0.2)
Vision problem	1.1 (1.1-1.1)	1.1 (1.1-1.1)	0.8 (0.8-0.9)	0.0 (0.0-0.0)
Hearing problem	0.9 (0.9-0.9)	1.0 (1.0-1.0)	-0.2 (-0.2--0.1)	0.0 (0.0-0.1)
Migraine	1.0 (1.0-1.0)	1.3 (1.2-1.5)	-0.9 (-1.3--0.6)	0.3 (0.1-0.5)
Epilepsy	3.4 (3.3-3.4)	3.5 (3.3-3.6)	12.3 (12.1-12.4)	1.2 (1.1-1.3)
Parkinson's disease	2.7 (2.7-2.7)	2.7 (2.5-2.9)	5.3 (5.1-5.4)	0.2 (0.1-0.3)
Multiple sclerosis	2.8 (2.8-2.9)	1.4 (1.1-1.7)	9.0 (8.6-9.3)	-0.2 (-0.4-0.0)
Neuropathies	1.3 (1.3-1.3)	1.5 (1.4-1.5)	1.8 (1.7-1.9)	0.2 (0.1-0.2)
Mental disorders	3.2 (3.2-3.3)	6.4 (6.3-6.5)	7.4 (7.4-7.5)	2.1 (2.0-2.1)
