# Supplementary material for: Analysis of mortality metrics associated with a comprehensive range of disorders in Denmark, 2000 to 2018: A population-based cohort study
Source: PLoS Med. 2022 Jun 16;19(6):e1004023. doi: 10.1371/journal.pmed.1004023 (PMC9202944; doi:10.1371/journal.pmed.1004023)
Supplement: S7 Table — Estimates are not shown if they are based on less than 100 individuals diagnosed or less than 20 deaths; for LYLs, estimates are not shown if there were not enough individuals at older ages of follow-up. LYLs, life years lost; MRR, mortality rate ratio. (PDF) [file pmed.1004023.s009.pdf]

**Analysis of mortality metrics associated with a comprehensive range of disorders in Denmark, 2000-2018: A population-based cohort study (Supporting information – S7 Table)**

S7 Table. Number of females and males diagnosed, and sex-specific mortality rate ratios and Life Years Lost for all causes of death for 39 selected conditions covering 10 broad categories. Estimates are not shown if they are based on less than 100 individuals diagnosed or less than 20 deaths; for Life Years Lost, estimates are not shown if there were not enough individuals at older ages of follow-up.

| Disorder | Sex | N | Age at diagnosis | Deaths | Age at death | MRR | LYL |
|---|---|---|---|---|---|---|---|
| Circulatory system | Females | 710669 | 69.0 (57.0-79.0) | 315712 | 84.1 (76.4-90.0) | 2.8 (2.8-2.8) | 3.8 (3.8-3.8) |
| Circulatory system | Males | 720372 | 64.5 (54.3-74.0) | 314148 | 79.0 (70.7-85.6) | 3.0 (3.0-3.0) | 3.8 (3.8-3.9) |
| Hypertension | Females | 414622 | 69.4 (58.0-78.9) | 157974 | 84.1 (76.6-89.9) | 1.7 (1.7-1.7) | 2.6 (2.6-2.6) |
| Hypertension | Males | 363256 | 65.6 (56.1-74.4) | 140784 | 78.6 (70.6-85.2) | 1.9 (1.8-1.9) | 3.2 (3.1-3.2) |
| Dislipidemia | Females | 123511 | 66.6 (57.0-75.3) | 35596 | 80.9 (73.4-87.0) | 1.4 (1.4-1.5) | 2.2 (2.1-2.3) |
| Dislipidemia | Males | 156600 | 63.3 (54.2-71.7) | 48615 | 76.6 (69.1-83.2) | 1.4 (1.4-1.4) | 2.1 (2.0-2.2) |
| Ischemic heart disease | Females | 211909 | 71.2 (60.1-80.6) | 112020 | 84.5 (77.5-90.0) | 1.9 (1.9-1.9) | 3.9 (3.8-3.9) |
| Ischemic heart disease | Males | 291525 | 65.6 (55.9-74.7) | 138837 | 79.5 (71.9-85.7) | 1.9 (1.9-1.9) | 3.5 (3.4-3.5) |
| Atrial fibrillation | Females | 167032 | 77.8 (69.3-84.8) | 101459 | 86.1 (79.9-91.0) | 2.3 (2.3-2.3) | 4.2 (4.1-4.2) |
| Atrial fibrillation | Males | 195493 | 71.6 (62.5-79.5) | 103646 | 81.3 (74.2-87.1) | 2.2 (2.2-2.2) | 4.2 (4.1-4.2) |
| Heart failure | Females | 112980 | 79.5 (71.0-86.1) | 87372 | 85.3 (78.7-90.6) | 3.2 (3.2-3.2) | 6.3 (6.2-6.4) |
| Heart failure | Males | 138907 | 73.4 (64.1-81.1) | 95643 | 80.5 (73.2-86.5) | 3.4 (3.4-3.4) | 6.6 (6.6-6.7) |
| Peripheral artery occlusive disease | Females | 98110 | 71.8 (61.6-80.1) | 55553 | 82.2 (75.1-88.2) | 2.6 (2.5-2.6) | 6.3 (6.2-6.4) |
| Peripheral artery occlusive disease | Males | 127298 | 69.3 (60.6-76.7) | 71754 | 78.3 (71.1-84.3) | 2.6 (2.6-2.6) | 5.9 (5.8-6.0) |
| Stroke | Females | 174605 | 75.3 (63.7-83.4) | 109406 | 84.3 (77.1-89.8) | 2.8 (2.8-2.8) | 6.3 (6.2-6.3) |
| Stroke | Males | 183826 | 69.8 (59.6-78.5) | 108475 | 79.6 (71.7-85.8) | 2.7 (2.7-2.8) | 6.1 (6.1-6.2) |
| Endocrine system | Females | 351582 | 59.2 (44.4-73.2) | 109742 | 82.2 (73.8-88.4) | 1.9 (1.9-1.9) | 4.2 (4.1-4.2) |
| Endocrine system | Males | 253621 | 61.6 (50.0-72.0) | 101423 | 76.7 (68.3-83.8) | 2.2 (2.2-2.3) | 5.4 (5.3-5.4) |
| Diabetes Mellitus | Females | 147845 | 65.4 (52.4-76.2) | 64747 | 81.1 (72.8-87.5) | 2.2 (2.2-2.2) | 6.1 (6.0-6.2) |
| Diabetes Mellitus | Males | 184344 | 62.2 (51.2-72.0) | 80937 | 75.9 (67.6-83.0) | 2.4 (2.4-2.4) | 6.2 (6.1-6.2) |
| Thyroid disorder | Females | 217475 | 54.8 (40.7-70.2) | 50185 | 83.0 (75.0-89.0) | 1.4 (1.4-1.5) | 2.3 (2.2-2.4) |
| Thyroid disorder | Males | 48076 | 60.1 (46.9-71.7) | 14016 | 79.4 (71.1-85.7) | 1.5 (1.5-1.5) | 2.9 (2.7-3.0) |
| Gout | Females | 14364 | 74.4 (62.5-82.9) | 7364 | 84.8 (77.8-90.3) | 2.0 (2.0-2.0) | 4.9 (4.7-5.0) |
| Gout | Males | 39675 | 64.4 (52.0-75.2) | 15351 | 79.0 (70.6-85.6) | 2.0 (2.0-2.0) | 4.7 (4.6-4.8) |
| Pulmonary system and allergy | Females | 357961 | 51.9 (25.9-69.8) | 101810 | 79.2 (71.6-85.7) | 2.8 (2.8-2.8) | 7.6 (7.5-7.6) |
| Pulmonary system and allergy | Males | 332384 | 48.9 (10.9-69.1) | 100834 | 77.9 (70.2-84.0) | 2.7 (2.7-2.7) | 7.4 (7.4-7.5) |
| Chronic pulmonary disease | Females | 289244 | 57.0 (28.3-72.1) | 97352 | 79.3 (71.8-85.6) | 3.0 (3.0-3.0) | 8.3 (8.2-8.3) |
| Chronic pulmonary disease | Males | 278786 | 53.9 (9.2-71.2) | 96928 | 78.1 (70.5-84.1) | 2.9 (2.9-2.9) | 8.3 (8.2-8.3) |
| Allergy | Females | 94266 | 36.1 (21.6-51.8) | 6660 | 77.1 (65.7-86.4) | 1.1 (1.1-1.1) | 0.3 (0.1-0.5) |
| Allergy | Males | 76473 | 31.0 (13.1-49.0) | 5740 | 72.1 (61.9-81.0) | 1.1 (1.1-1.1) | 0.3 (0.1-0.6) |
| Gastrointestinal system | Females | 243859 | 64.8 (50.3-76.6) | 93039 | 82.6 (73.2-89.1) | 1.9 (1.9-1.9) | 5.3 (5.3-5.4) |
| Gastrointestinal system | Males | 230474 | 61.3 (47.7-72.6) | 92989 | 76.2 (65.3-84.2) | 2.2 (2.2-2.3) | 6.8 (6.7-6.9) |
| Ulcer/chronic gastritis | Females | 98118 | 69.8 (55.0-80.4) | 52809 | 83.2 (74.8-89.4) | 2.2 (2.1-2.2) | 6.4 (6.3-6.4) |
| Ulcer/chronic gastritis | Males | 96094 | 65.1 (51.9-76.0) | 51529 | 78.1 (68.6-85.1) | 2.3 (2.3-2.3) | 6.8 (6.7-6.9) |
| Chronic liver disease | Females | 27071 | 53.9 (41.4-64.2) | 12123 | 65.0 (56.2-74.1) | 6.8 (6.7-7.0) | 17.0 (16.9-17.2) |
| Chronic liver disease | Males | 43224 | 53.8 (43.4-63.3) | 23812 | 62.3 (54.4-70.2) | 8.5 (8.4-8.6) | 17.1 (16.9-17.2) |
| Inflamatory bowel disease | Females | 41040 | 43.3 (28.2-61.1) | 7081 | 79.5 (69.9-86.5) | 1.5 (1.5-1.6) | 3.7 (3.5-3.9) |
| Inflamatory bowel disease | Males | 34167 | 43.3 (28.4-59.8) | 6118 | 76.2 (66.3-83.3) | 1.4 (1.4-1.4) | 2.7 (2.4-2.9) |
| Diverticular disease of intestine | Females | 100084 | 69.6 (59.8-78.1) | 32456 | 85.1 (78.4-90.4) | 1.2 (1.2-1.2) | 1.2 (1.2-1.3) |
| Diverticular disease of intestine | Males | 79933 | 67.2 (57.3-75.2) | 24220 | 81.5 (74.3-87.1) | 1.2 (1.2-1.3) | 1.3 (1.2-1.4) |
| Urogenital system | Females | 49210 | 73.1 (56.9-82.5) | 27716 | 81.5 (72.7-87.9) | 4.2 (4.1-4.2) | 10.4 (10.3-10.5) |
| Urogenital system | Males | 205916 | 70.7 (62.5-78.3) | 111750 | 81.8 (74.9-87.3) | 1.6 (1.6-1.6) | 3.1 (3.0-3.1) |
| Chronic kidney disease | Females | 49199 | 73.1 (56.9-82.5) | 27713 | 81.5 (72.7-87.9) | 4.2 (4.1-4.2) | 10.4 (10.3-10.5) |
| Chronic kidney disease | Males | 70090 | 72.7 (60.7-81.1) | 43412 | 79.4 (70.9-85.8) | 4.2 (4.2-4.2) | 9.0 (8.9-9.0) |
| Prostate disorders | Females | - | - | - | - | - | - |
| Prostate disorders | Males | 150437 | 70.9 (63.5-77.9) | 79099 | 83.0 (77.1-88.1) | 1.2 (1.2-1.2) | 0.8 (0.7-0.8) |
| Musculoskeletal system | Females | 284567 | 67.4 (54.7-77.6) | 102756 | 83.2 (75.3-89.3) | 1.7 (1.7-1.7) | 3.7 (3.6-3.7) |
| Musculoskeletal system | Males | 110447 | 65.9 (51.5-75.5) | 45317 | 78.1 (69.6-84.8) | 2.2 (2.2-2.2) | 6.3 (6.2-6.4) |
| Connective tissue disorders | Females | 118495 | 58.3 (39.6-72.5) | 33790 | 82.4 (74.6-88.3) | 1.5 (1.4-1.5) | 3.2 (3.1-3.3) |
| Connective tissue disorders | Males | 58813 | 59.4 (39.3-71.8) | 17599 | 79.0 (71.0-85.1) | 1.5 (1.4-1.5) | 3.5 (3.4-3.7) |
| Osteoporosis | Females | 189307 | 71.6 (62.2-80.3) | 79543 | 83.7 (75.9-89.6) | 1.8 (1.8-1.8) | 3.6 (3.6-3.7) |
| Osteoporosis | Males | 57059 | 70.7 (61.5-78.7) | 30435 | 77.7 (69.1-84.6) | 3.0 (2.9-3.0) | 7.3 (7.2-7.4) |
| Hematological system | Females | 159072 | 73.0 (51.8-83.2) | 90777 | 83.6 (74.4-89.9) | 3.3 (3.3-3.3) | 9.7 (9.7-9.8) |
| Hematological system | Males | 123818 | 72.6 (60.8-81.3) | 83958 | 79.1 (69.9-86.0) | 4.5 (4.5-4.5) | 9.6 (9.6-9.7) |
| HIV/AIDS | Females | 1731 | 33.5 (27.8-40.8) | 241 | 48.3 (40.9-58.7) | 5.0 (4.4-5.7) | - |
| HIV/AIDS | Males | 4800 | 39.7 (32.4-48.5) | 981 | 55.2 (45.9-64.5) | 3.4 (3.2-3.7) | 9.8 (8.0-10.9) |
| Anemias | Females | 157600 | 73.2 (52.5-83.3) | 90612 | 83.6 (74.5-89.9) | 3.3 (3.3-3.3) | 9.7 (9.6-9.7) |
| Anemias | Males | 119371 | 73.3 (62.4-81.6) | 83165 | 79.2 (70.2-86.1) | 4.5 (4.5-4.5) | 9.5 (9.4-9.5) |
| Cancers | Females | 331406 | 66.4 (55.4-76.2) | 176621 | 76.2 (66.6-84.4) | 4.6 (4.5-4.6) | 10.4 (10.3-10.4) |
| Cancers | Males | 308632 | 68.7 (60.1-76.2) | 187822 | 75.0 (66.5-82.3) | 5.4 (5.4-5.5) | 9.4 (9.4-9.5) |
| Neurological system | Females | 706806 | 66.0 (47.6-76.8) | 238408 | 85.9 (78.5-91.3) | 1.1 (1.1-1.2) | 1.4 (1.4-1.4) |
| Neurological system | Males | 577074 | 63.7 (48.0-73.9) | 204341 | 81.2 (72.8-87.4) | 1.3 (1.3-1.3) | 2.2 (2.1-2.2) |
| Vision problem | Females | 308721 | 75.2 (68.2-81.2) | 141316 | 86.8 (80.8-91.6) | 1.0 (1.0-1.0) | 0.6 (0.5-0.6) |
| Vision problem | Males | 202828 | 73.5 (65.7-79.8) | 94117 | 83.6 (77.2-88.7) | 1.2 (1.2-1.2) | 1.3 (1.2-1.3) |
| Hearing problem | Females | 235022 | 71.9 (57.4-80.9) | 105056 | 88.6 (82.7-93.1) | 0.9 (0.9-0.9) | 0.0 (0.0-0.1) |
| Hearing problem | Males | 242408 | 67.2 (55.5-75.7) | 97150 | 83.7 (77.1-89.0) | 0.9 (0.9-0.9) | -0.2 (-0.3--0.2) |
| Migraine | Females | 57949 | 37.6 (25.4-49.1) | 2815 | 71.3 (59.1-82.8) | 1.0 (1.0-1.1) | -0.6 (-0.9--0.2) |
| Migraine | Males | 23193 | 32.3 (14.6-47.1) | 1262 | 69.2 (57.0-78.6) | 1.0 (0.9-1.0) | -0.8 (-1.4--0.3) |
| Epilepsy | Females | 56328 | 44.7 (19.2-66.8) | 18390 | 75.8 (63.4-84.8) | 3.3 (3.2-3.3) | 13.3 (13.1-13.5) |
| Epilepsy | Males | 62199 | 45.6 (19.6-64.8) | 22554 | 70.1 (58.2-79.7) | 3.5 (3.4-3.5) | 13.7 (13.5-13.9) |
| Parkinson's disease | Females | 13386 | 75.7 (68.5-81.5) | 8966 | 83.1 (77.9-87.6) | 2.7 (2.6-2.8) | 5.7 (5.6-5.9) |
| Parkinson's disease | Males | 18079 | 74.3 (66.9-80.3) | 11878 | 81.0 (75.5-85.6) | 2.7 (2.7-2.8) | 5.3 (5.2-5.4) |
| Multiple sclerosis | Females | 13989 | 43.5 (34.0-53.2) | 2691 | 67.2 (58.6-76.2) | 2.8 (2.7-2.9) | 8.7 (8.3-9.0) |
| Multiple sclerosis | Males | 6928 | 45.6 (35.4-55.2) | 1921 | 66.1 (57.0-74.2) | 2.8 (2.7-2.9) | 8.9 (8.5-9.4) |
| Neuropathies | Females | 177167 | 53.1 (41.1-66.2) | 28844 | 80.1 (68.9-88.3) | 1.1 (1.1-1.1) | 1.0 (0.9-1.1) |
| Neuropathies | Males | 140769 | 54.7 (42.3-66.8) | 31321 | 74.2 (64.5-82.8) | 1.5 (1.4-1.5) | 3.1 (3.0-3.2) |
| Mental disorders | Females | 591588 | 43.0 (23.0-70.4) | 166916 | 83.8 (73.9-89.8) | 3.0 (2.9-3.0) | 8.3 (8.3-8.4) |
| Mental disorders | Males | 537389 | 39.7 (20.1-61.3) | 151281 | 75.2 (62.2-84.3) | 3.9 (3.8-3.9) | 10.8 (10.7-10.8) |
