# Supplementary material for: Analysis of mortality metrics associated with a comprehensive range of disorders in Denmark, 2000 to 2018: A population-based cohort study
Source: PLoS Med. 2022 Jun 16;19(6):e1004023. doi: 10.1371/journal.pmed.1004023 (PMC9202944; doi:10.1371/journal.pmed.1004023)
Supplement: S1 Fig — ICD-10, International Classification of Diseases-10th Revision. (PDF) [file pmed.1004023.s010.pdf]

The Danish Atlas of Disease Mortality

Analysis of mortality metrics associated with a comprehensive range of disorders in Denmark, 2000-2018

Summary statement

A total of 1,128,977 persons living in Denmark in 2000-2018 were diagnosed for the first time with 'Mental disorders' (ICD-10: F00-F99) in a hospital in 1995-2018. The median age at diagnosis was 41.4 years, while 25% of the diagnosed were younger than 21.6 and 25% were older than 65.6 years at time of diagnosis. Among the diagnosed, 318,197 persons died in 2000-2018, and the median age at time of death was 80.2 years (25% were younger than 67.5 years and 25% were older than 87.7 years). On average, mortality rates were 3.35 times higher among the diagnosed compared to those of same age and sex without that diagnosis (MRR = 3.35 (95% CI: 3.34, 3.37)), and the average reduction in life expectancy after diagnosis was 9.50 years compared to the entire Danish population of same age and sex (LYL = 9.50 (95% CI: 9.46, 9.54)).

Note: The estimate of life expectancy is based on the assumption that the diagnosed will experience the mortality rates of the diagnosed during the entire life (after diagnosis), which might be plausible for chronic disorders but not for acute ones.

Summaries split by sex

Sex	Diagnosed	Age at Diagnosis [Median (IQR)]	Deaths	Age at Death [Median (IQR)]	Mortality Rate Ratio	Life Years Lost
Persons	1,128,977	41.4 (21.6 - 65.6)	318,197	80.2 (67.5 - 87.7)	3.35 (3.34 - 3.37)	9.50 (9.46 - 9.54)
Men	537,389	39.7 (20.1 - 61.3)	151,281	75.2 (62.2 - 84.3)	3.85 (3.83 - 3.87)	10.79 (10.72 - 10.85)
Women	591,588	43.0 (23.0 - 70.4)	166,916	83.8 (73.9 - 89.8)	2.95 (2.93 - 2.97)	8.33 (8.27 - 8.39)

Age at diagnosis

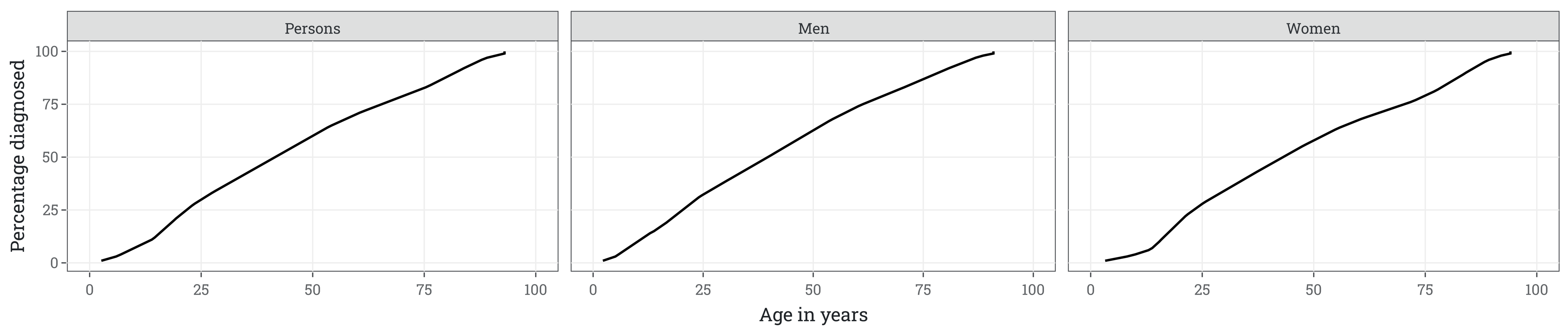

Age at death

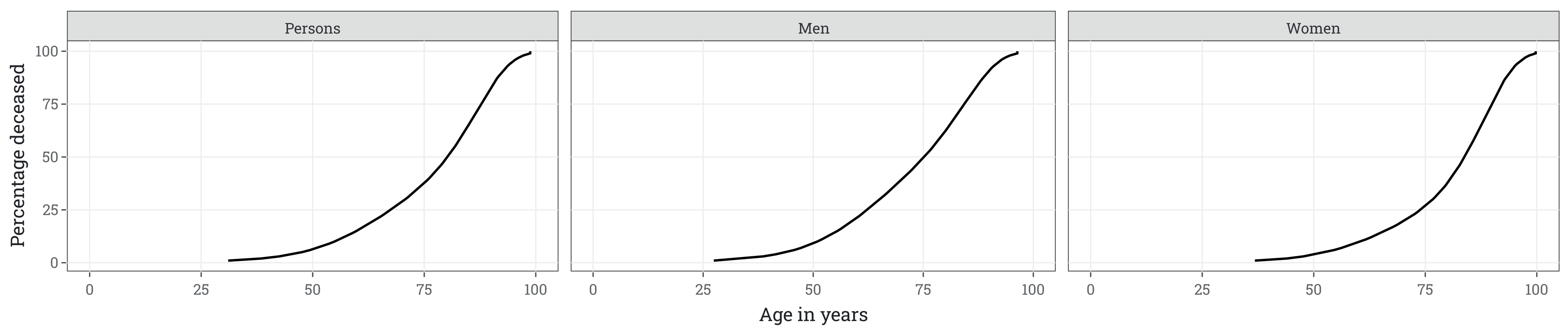

Incidence rate given age

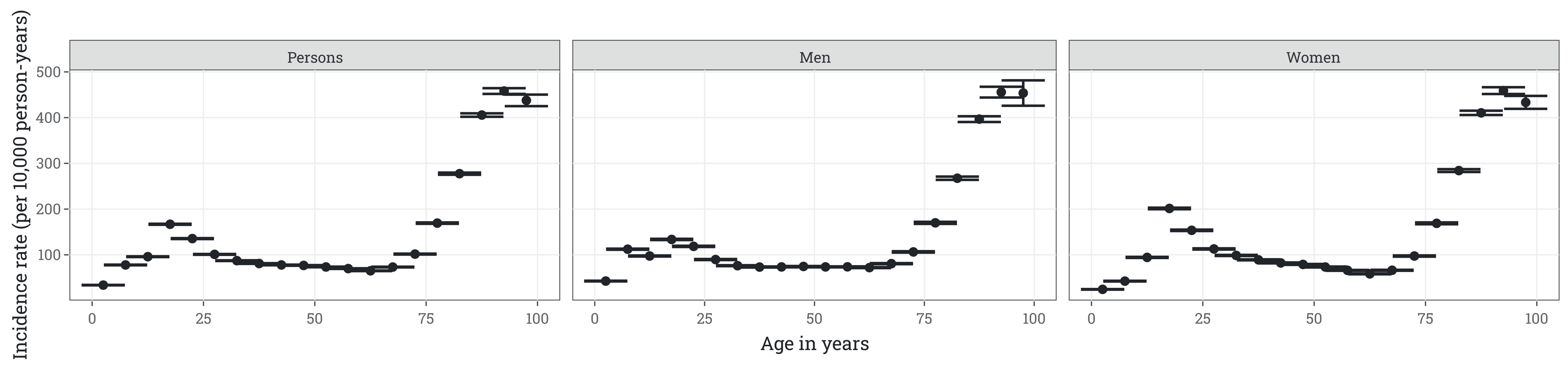

Click on a point for more information

Mortality rates and mortality rate ratio given age

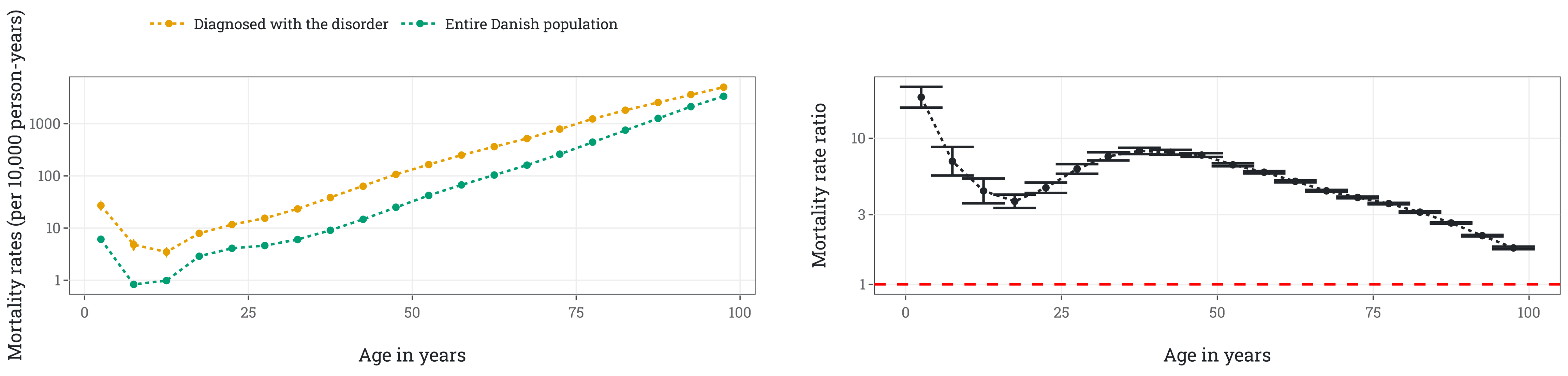

Click on a point for more information

persons

Mortality rate ratio given time since diagnosis

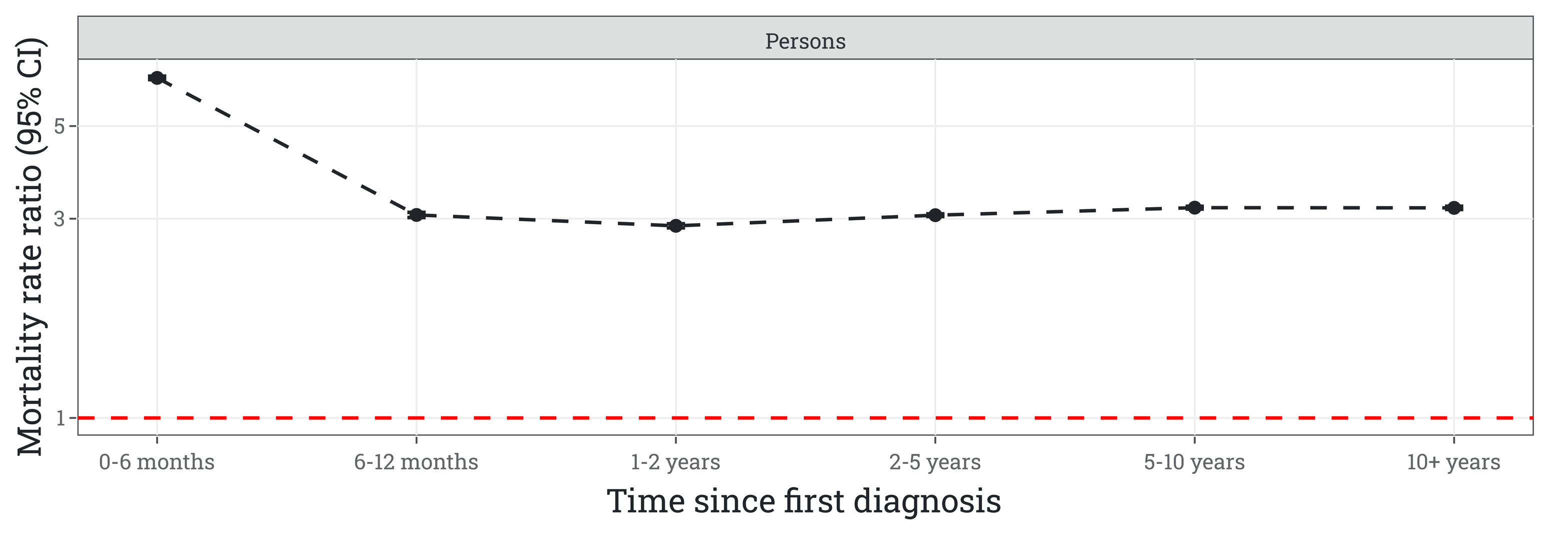

Click on a point for more information

Expected remaining life expectancy at a given age

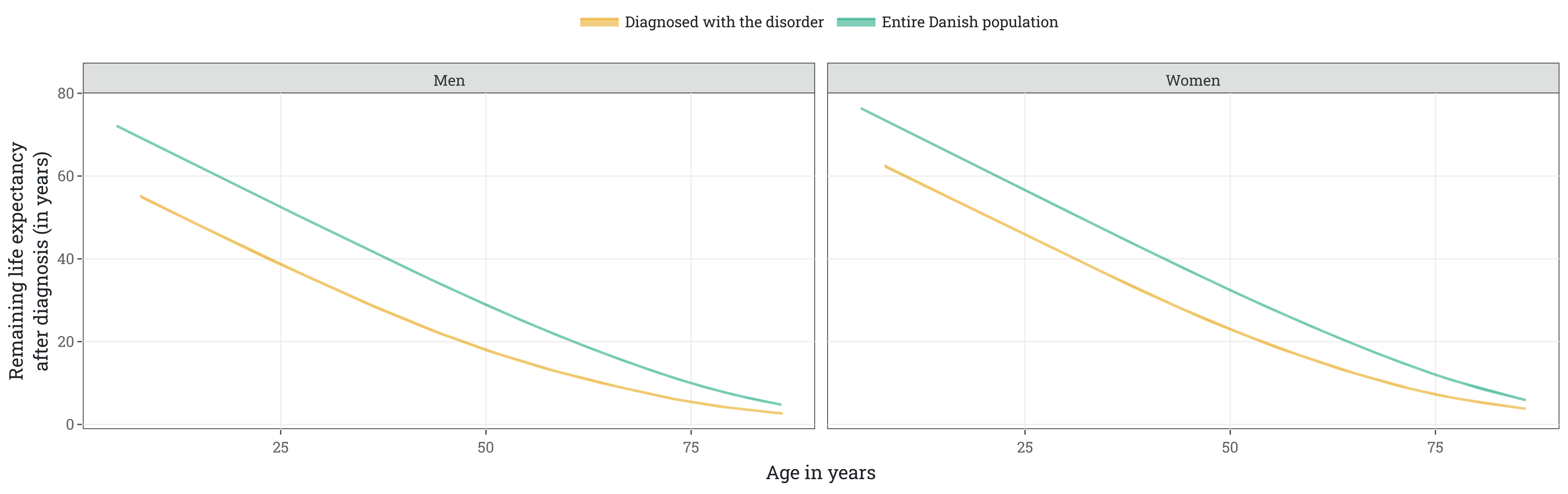

Survival curves

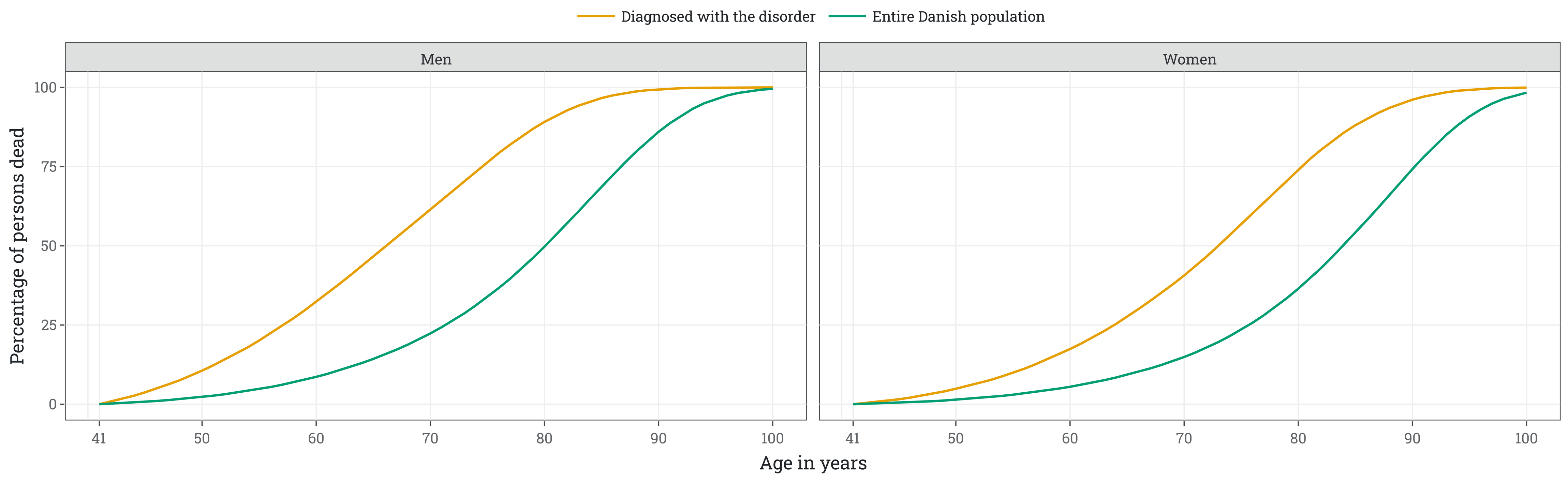

Starting age

41 years (50th percentile)

Estimates for all-causes of cause-specific mortality:

All causes
