# Supplementary material for: Analysis of mortality metrics associated with a comprehensive range of disorders in Denmark, 2000 to 2018: A population-based cohort study
Source: PLoS Med. 2022 Jun 16;19(6):e1004023. doi: 10.1371/journal.pmed.1004023 (PMC9202944; doi:10.1371/journal.pmed.1004023)
Supplement: S2 Fig — The red line indicates equal mortality in the 2 groups (MRR of 1). All estimates are adjusted for age, sex, and birth date. Estimates are available in S5 Table and on Open Science Framework [16]. MRR, mortality rate ratio. (PDF) [file pmed.1004023.s011.pdf]

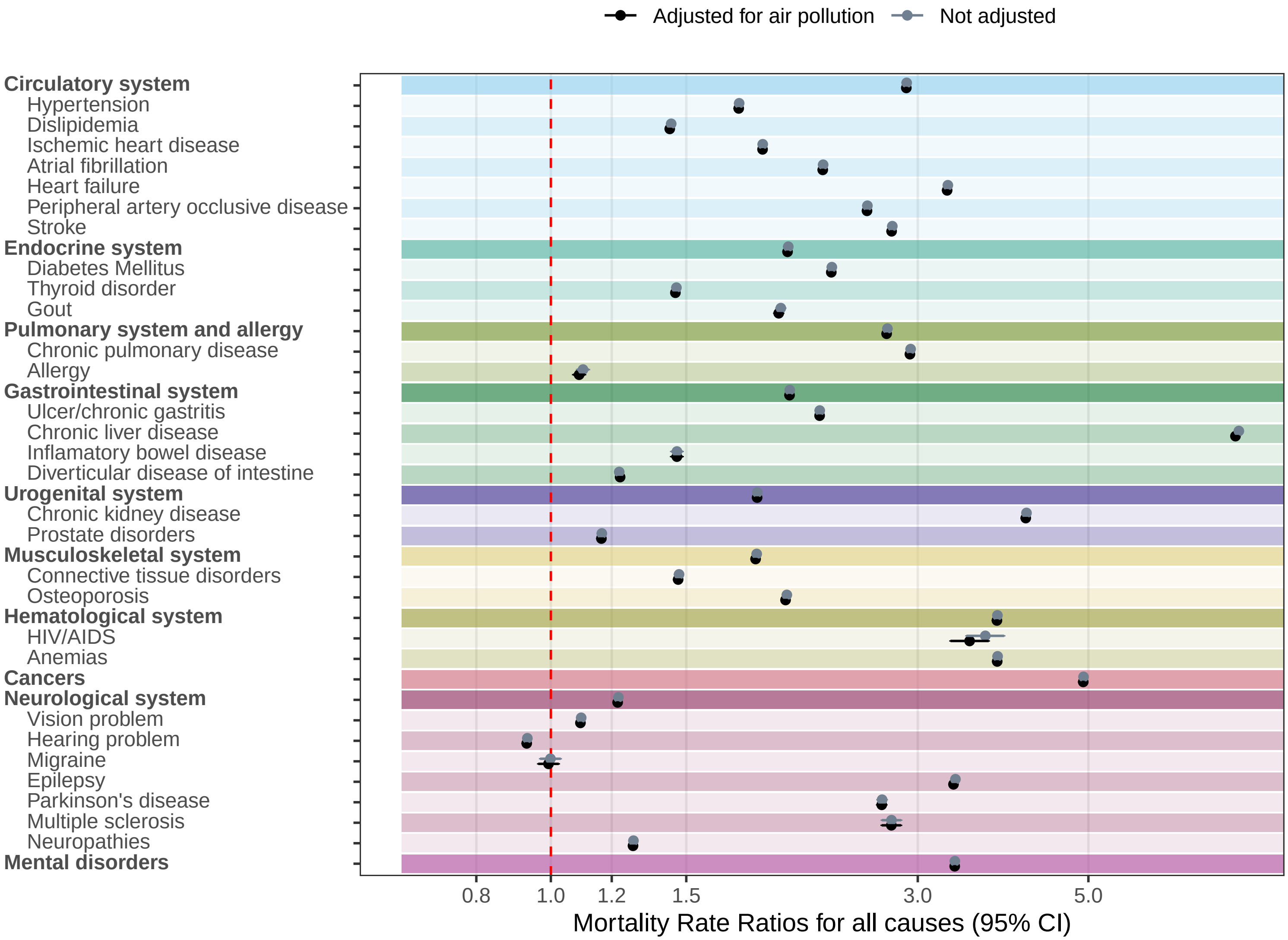

Adjusted for air pollution
Not adjusted
Circulatory system
Hypertension
Dislipidemia
Ischemic heart disease
Atrial fibrillation
Heart failure
Peripheral artery occlusive disease
Stroke
Endocrine system
Diabetes Mellitus
Thyroid disorder
Gout
Pulmonary system and allergy
Chronic pulmonary disease
Allergy
Gastrointestinal system
Ulcer/chronic gastritis
Chronic liver disease
Inflamatory bowel disease
Diverticular disease of intestine
Urogenital system
Chronic kidney disease
Prostate disorders
Musculoskeletal system
Connective tissue disorders
Osteoporosis
Hematological system
HIV/AIDS
Anemias
Cancers
Neurological system
Vision problem
Hearing problem
Migraine
Epilepsy
Parkinson's disease
Multiple sclerosis
Neuropathies
Mental disorders
0.8
1.0
1.2
1.5
3.0
5.0
Mortality Rate Ratios for all causes (95% CI)
