# Supplementary figures and images for: Analysis of mortality metrics associated with a comprehensive range of disorders in Denmark, 2000 to 2018: A population-based cohort study
Source: PLoS Med. 2022 Jun 16;19(6):e1004023. doi: 10.1371/journal.pmed.1004023 (PMC9202944; doi:10.1371/journal.pmed.1004023)

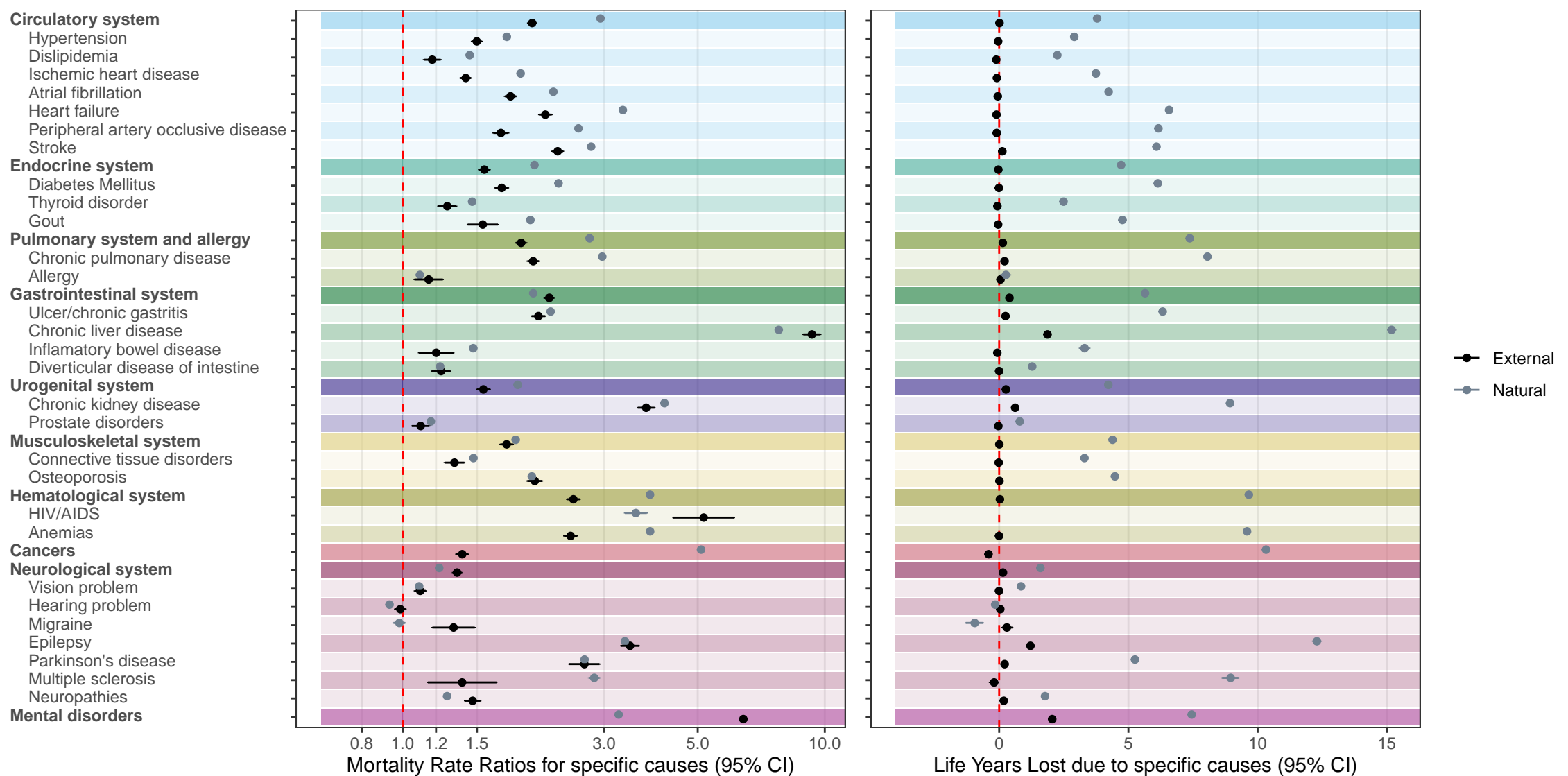

Supplement: S3 Fig — The red line indicates equal mortality in the 2 groups (MRR of 1; LYLs of 0). Estimates are not shown if they are based on less than 100 individuals diagnosed or less than 20 deaths; for LYLs, estimates are not shown if there were not enough individuals at older ages of follow-up. Estimates are available in S6 Table and on Open Science Framework [16]. LYLs, life years lost; MRR, mortality rate ratio. (PDF) [file pmed.1004023.s012.pdf]

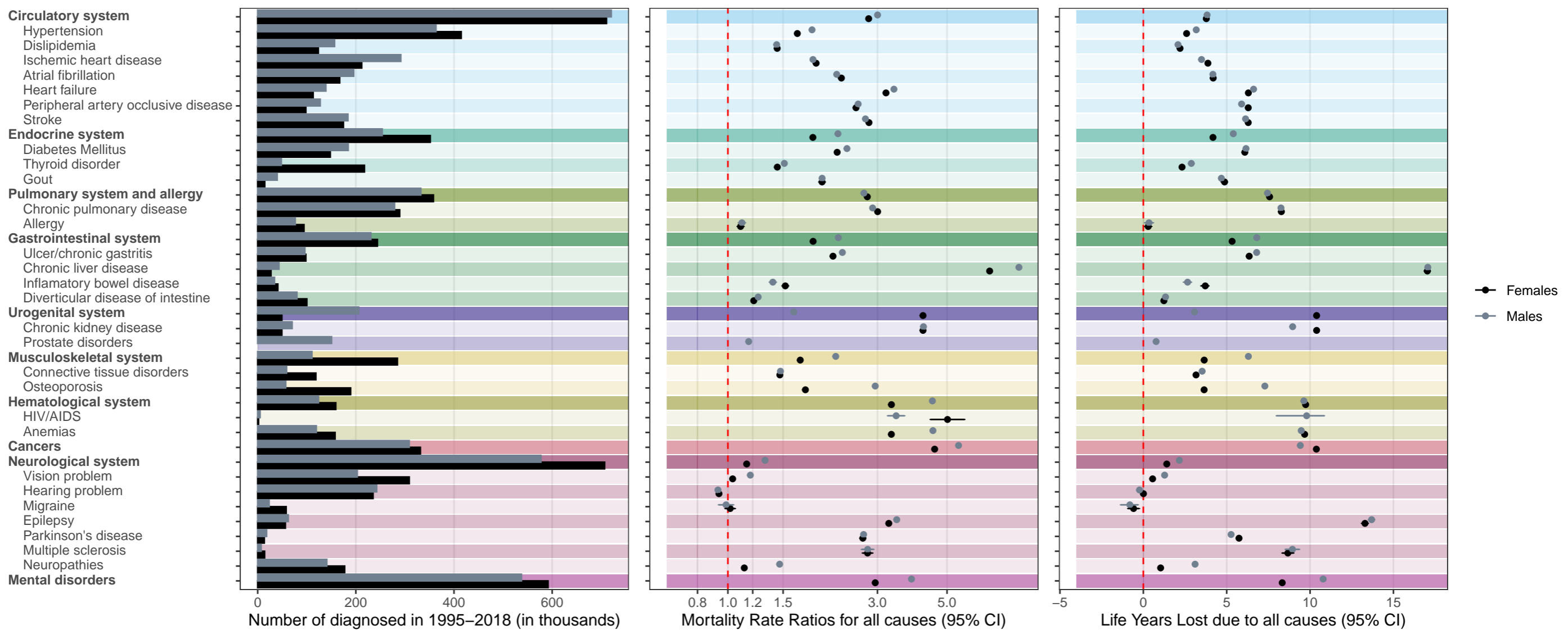

Supplement: S4 Fig — The red line indicates equal mortality in the 2 groups (MRR of 1; LYLs of 0). Estimates are not shown if they are based on less than 100 individuals diagnosed or less than 20 deaths; for LYLs, estimates are not shown if there were not enough individuals at older ages of follow-up. Estimates are available in S7 Table and on Open Science Framework [16]. LYLs, life years lost; MRR, mortality rate ratio. (PDF) [file pmed.1004023.s013.pdf]
