# Supplementary material for: Analysis of mortality metrics associated with a comprehensive range of disorders in Denmark, 2000 to 2018: A population-based cohort study
Source: PLoS Med. 2022 Jun 16;19(6):e1004023. doi: 10.1371/journal.pmed.1004023 (PMC9202944; doi:10.1371/journal.pmed.1004023)
Supplement: Attachment — Submitted filename: AtlasMortality_Cover letter and response to reviewers.pdf [file pmed.1004023.s014.pdf]

Dear Dr Moyer,

Many thanks for allowing us the opportunity to revise and resubmit this manuscript. The reviewers have provided us with many constructive suggestions. We have addressed each reviewer point below. Two versions of the revised manuscript have been uploaded (a 'clean' version, and one with track changes showing corrections). The page and line numbers refer to the version with track changes.

Editorial comments

1. Title: Please revise your title according to PLOS Medicine's style. Your title must be nondeclarative and not a question. It should begin with main concept if possible. "Effect of" should be used only if causality can be inferred, i.e., for an RCT. Please place the study design ("A randomized controlled trial," "A retrospective study," "A modelling study," etc.) in the subtitle (ie, after a colon).

RESPONSE: We have changed the title to "An atlas of mortality metrics associated with a comprehensive range of disorders. A Danish cohort study".

2. Line numbers: Please include line numbers running continuously throughout the manuscript with the revised version.

RESPONSE: We have now added line numbers running continuously.

3. Abstract: Methods and Findings: Please include a few examples or broad summary of some of the 39 general medical conditions discussed. For example, as described in the Methods: "...ten broad categories: circulatory, endocrine, pulmonary, gastrointestinal, urogenital, musculoskeletal, hematologic, mental, and neurologic conditions and cancer…"

RESPONSE: We have now provided the list of broad categories in the abstract (page 4, lines 96-98)

4. Abstract: Methods and Findings: Please mention which conditions correspond with these MRRs/LYLs given as examples: "For these 37 disorders, MRRs ranged from 1.09 (95%CI: 1.09-1.10) to 7.85 (7.77-7.93), while LYLs ranged from 0.31 (0.14-0.47) years (~16 weeks) to 17.05 (16.95-17.15) years."

RESPONSE: We have now added the conditions: "For these 37 disorders, MRRs ranged from 1.09 (95%CI: 1.09-1.10) <u>for vision problems</u> to 7.85 (7.77-7.93) <u>for chronic liver disease</u>, while LYLs ranged from 0.31 (0.14-0.47) years (~16 weeks) <u>for allergy</u> to 17.05 (16.95-17.15) years <u>for chronic liver disease</u>" (page 4, lines 102-103).

5. Abstract: Methods and Findings: In the last sentence of the Abstract Methods and Findings section, please describe the main limitation(s) of the study's methodology.

RESPONSE: We have now added "The association between the different disorders and mortality could be confounded by underlying risk factors associated both with the disorder and mortality" (page 4, lines 104-105).

6. Abstract: Conclusions: Please address the study implications without overreaching what can be concluded from the data; the phrase "In this study, we observed ..." may be useful. Please avoid or temper assertions of primacy ("To the best of our knowledge, this atlas is the first....").

RESPONSE: We have now modified the first sentence of the conclusions: "<u>In this study</u>, we show <u>estimates of incidence, age-of-onset, age-of-death and mortality metrics</u> (both MRRs and LYLs) for a comprehensive range of disorders" (page 4, lines 109-110).

7. Author summary: At this stage, we ask that you include a short, non-technical Author Summary of

your research to make findings accessible to a wide audience that includes both scientists and non-scientists. The Author Summary should immediately follow the Abstract in your revised manuscript. This text is subject to editorial change and should be distinct from the scientific abstract. Please see our author guidelines for more information: https://journals.plos.org/plosmedicine/s/revising-your-manuscript#loc-author-summary

RESPONSE: We have provided a short, non-technical summary of the study in page 5.

8. In text citations: Please place reference citations within square brackets, placed before the sentence punctuation, for example [1,2]. Where multiple references are listed, please do not include spaces within brackets.

RESPONSE: We have updated the citation style. Notice that we have not used track changes for this change.

9. Introduction: "A protocol was pre-registered before having access to the data and a webpage has been designed to visualize all results (https://nbepi.com/atlas)." Please move this information to the Methods.

RESPONSE: We have moved this information to the methods section (page 7, lines 194-195).

10. Methods: "We designed a population-based cohort study including all 7,378,598 persons living in Denmark at any point between January 1, 2000 and December 31, 2018" Are more recent data available?

RESPONSE: Unfortunately, not. The Danish Health Authority changed the format of the hospital register in 2019, and there is currently no access to updated data for research purposes. There is a temporary version of the updated hospital data made especially for COVID-19 projects, but unfortunately it does not cover the national population yet. It is expected that a final version will be made available for researchers towards the end of 2022. At that point, a new data application would be necessary, and it would take around an additional 15 months to have access to the data. We hope that our study can serve as a benchmark and stimulate future hypothesis generation (e.g. how has the COVID-19 epidemic influenced the wider pattern of mortality described in the current paper).

11. Methods: Statistical analysis: Please clarify if comorbidity status or level of education were taken into account in the analyses.

RESPONSE: Comorbidity is a topic that we are very familiar with, with recent highly cited papers on comorbidity within mental disorders (Plana-Ripoll et al. 2019) and between mental disorders and other conditions (Momen et al. 2020). We know that there were many individuals included in the current study who would have been diagnosed with more than one diagnosis. The computational load for adjusting for these factors would be considerable. We would have to temporally-order each disorder for each individual, and then examine a subset of several hundred thousand sets of different disorder combinations. Additionally, there would be several disorders extremely correlated, thus the set of comorbid conditions to adjust for should be different for each of the 1,803 disorders considered in the study. We did not have access to data regarding socio-economic characteristics, so we are not able to address this issue in the current study.

We have now clarified in the methods section: "Individuals with more than one diagnosis contributed information for each of their diagnoses; however, only information in relation to the specific disorder was considered in the estimates" (page 8, lines 235-237) and "The models did not adjust for socio-economic characteristics, as data at the individual-level could not be used" (page 10, lines 270-272). Additionally, we have stated in the discussion: "This study did not include information on remission or other comorbid disorders; the group of

individuals with a specific disorder can therefore be interpreted as persons who have had a diagnosis of the disorder regardless of whether they have other disorders or whether they have recovered afterwards" (page 15, lines 434-437).

12. Methods: Given Reviewer 1's comment about underlying social determinants of health, and the likelihood that these are geographically clustered, please explain if clustering by region or deprivation index was taken into account.

RESPONSE: We did not take into account the geographical clustering. Denmark is a relatively small and homogenous country with only 5.8 million individuals. Additionally, healthcare is universal and free for all residents. One of the reasons we did not use data aggregated in regions is because we had access to individual-level data on morbidity and mortality. By doing that, individuals were followed-up during the entire observation period regardless of whether they changed their home address.

13. Methods: Please include a copy of the pre-specified analysis plan for the study as a supporting information file.

RESPONSE: We have now provided a copy of the pre-specific analysis plan in S2 Text.

14. Methods: Please report your data according to GATHER (or according to the most relevant guideline for your study) and enclose a completed GATHER checklist as a supplementary document. Please add the following statement, or similar, to the Methods: "This study is reported as per the Guidelines for Accurate and Transparent Health Estimates Reporting (GATHER) guideline (S1 Checklist)." Please see http://gather-statement.org/ for more information.

RESPONSE: We have reported the data according to GATHER, have provided a checklist as supplementary material (S2 Table), and added the GATHER statement (page 10, lines 274-275).

15. Results: Please clarify which subchapters are being described here: "When looking within ICD chapters (Figure 5b), there is considerable heterogeneity, with MRRs ranging from 0.50 (0.38 to 0.64) to 152.31 (116.61 to 198.93); and LYLs ranging from -3.37 (-3.48 to -3.24) years to 26.95 (25.43 to 28.58) years."

RESPONSE: We have now specified the subchapters for all estimates: "When looking within ICD chapters (Figure 5b), there is considerable heterogeneity, with MRRs ranging from 0.50 (0.38 to 0.64)<u>) for ICD-10 code O83 (Other assisted single delivery; Chapter XV)</u> to 152.31 (116.61 to 198.93<u>) for P60 (Disseminated intravascular coagulation of newborn; Chapter XVI)</u>; and LYLs ranging from -3.37 (-3.48 to -3.24) <u>years for M23 (Internal derangement of knee; Chapter XIII)</u> to 26.95 (25.43 to 28.58) years <u>for C74 (Malignant neoplasm of adrenal gland; Chapter II)</u>" (page 12, lines 354-358).

16. Discussion: Please present and organize the Discussion as follows: a short, clear summary of the article's findings; what the study adds to existing research and where and why the results may differ from previous research; strengths and limitations of the study; implications and next steps for research, clinical practice, and/or public policy; one-paragraph conclusion.

RESPONSE: We have restructured the discussion according to the suggested topics.

17. Figure 1 and Figure 2: If possible, please also provide these data in table format.

RESPONSE: We have now provided the estimates in supplementary tables as well (S4 Table).

Reviewer #1:

The study authors have created a fantastic resource exploiting the fantastic ability in Denmark to link health service records to death. This will be a resource for many users and particularly for burden of disease estimation. I only opened the website after reading the full paper and found it a much greater resource than I could have imagined from just reading the paper. It seems that you are 'under-selling' what you are giving the world! The least I would suggest you can do is provide an example for one particular condition showing all the results that you can find in the tool after clicking on that specific disease. I have a lot of more specific comment below, largely asking for more precise detail but a few more conceptual things and limitations that you have not spun out in enough detail.

RESPONSE: Thank you for your positive comment and helpful feedback. We have now included an additional figure in the appendix with an overview of all estimates for one specific disorder (S1 Figure).

Specific comments:

1. Abstract: I would not call life expectancy an 'absolute-risk mortality metric'

   RESPONSE: We have changed the sentence to "The provision of different types of mortality metrics" (page 4, line 83).

2. Abstract: how did you compute the 7.4 million persons living in Denmark 2000-2018? From a statement a bit further it seems that you conceptualise is as "anyone who lived in Denmark for some part of all of 2000-2018'

   RESPONSE: This is correct. While some individuals might have lived in Denmark for the entire 19-year period, many others might have lived there for a shorter period (if they were born or immigrated after 1 January 2000, or if they died or emigrated before 31 December 2018). This is explained in detail in the methods section, but is abbreviated in the abstract due to space constraints. However, we have updated the abstract to make it clearer "In a population-based cohort of all 7,378,598 persons living in Denmark <u>at some point between</u> 2000 and 2018" (page 4, lines 89-90).

3. Abstract: you do not define LYL

   RESPONSE: LYL is spelled in full as 'life years lost' in the first appearance (page 4, line 94).

4. Introduction: The statement "When looking at life expectancy, the Global Burden of Disease (GBD) studies measure Years of Life Lost (YLLs),3 which estimates the potential years of life lost." suggests that YLLs are a measure of life expectancy which it is not

   RESPONSE: We have replaced 'life expectancy' by 'premature mortality' (page 6, line 149).

5. Page 7 line 2: what do you mean with 'admissions to ….outpatient facilities'? Do you mean e.g. day surgeries or also consultations with medical specialists?

   RESPONSE: Sorry about that. We mean 'visits' to medical specialists (not admissions to hospital). We have updated the sentence to: "…all admissions to hospital in<u>patient facilities</u> and <u>visits to</u> outpatient facilities <u>(including visits to medical specialists)</u>, as well as emergency departments…" (page 8, lines 207-208).

6. Page 8: you estimate your MRRs and difference in average life expectancy against population all-cause mortality rates (although I am a little confused by the contradicting statement on page

10: "mortality rates after diagnosis remained higher for those with a diagnosis of any of the disorders, compared to those without the diagnosis"). These population mortality rates also include the deaths from any condition of interest. Conceptually, I think you are using a counterfactual approach: "if a person had not become a case of a disease, how different would this person's risk of death and remaining life expectancy have been?". To do that correctly you would need to contrast people with a disorder with the rest of the population without the disorder. Most disorders will be a rare enough reason for death to make such comparisons a reasonable proxy for a true RR or difference in life expectancy but you also include disorders such as IHD and stroke that are highly prevalent/incident at oldest ages and then it would not be a very good proxy.

RESPONSE: This is a very important comment, and there are several things to clarify. First, it is important to differentiate between health disorders (identified through hospital contacts during lifetime) and causes of death (identified through death certificates at time of death). All MRRs and life expectancy are estimated for each health disorder considering (i) all-cause mortality (all deaths regardless of the underlying cause of death); and (ii) cause-specific mortality (all deaths divided into natural or external causes of death). Thus, for one specific health disorder (e.g. mental disorders), MRRs and life expectancy were estimated for those diagnosed with a mental disorder regardless of whether a mental disorder was considered as the underlying cause of death or not. This is one of the main strengths of the life years lost method.

Second, MRRs compare mortality rates among individuals with a diagnosis of a specific disease and individuals without that particular diagnosis, as the reviewer suggests. This is a widely used and easy to follow mortality metric. However, the recently introduced life years lost address a different (and even more interesting) question – it compares remaining life expectancy for (a) individuals with a diagnosis, compared to (b) the general population of same age and sex. The main reason to compare those with a given disease to the general population – and not to persons without the disease – is that the number of LYL at a given age, e.g. 45 years, is estimated using mortality rates at ages 45 years and beyond. By choosing persons without the disease as a comparison group, we would assume that someone who has not experienced the disease at age 45, would remain free of the disease until death. We have explained this issue in our methodological paper about the LYL measure (Plana-Ripoll et al. Plos One 2020). Although it might seem problematic to include persons with a disease in both the diseased and reference groups, this is analogous to widely used (and classic) standardized mortality ratios (SMRs), which compare mortality in a group of persons to the one in the general population. In any case, differences in life expectancy would be even larger if the comparison group were persons without the disease. We have now included this explanation in S1 Text where we describe the life years lost method in detail and provide a worked example (as suggested in comment #1 from Reviewer 3).

7. Results: when you present prevalence of aggregates of the 39 selected disorders, are you conceptualizing that as an individual experiencing any of the more specific disorders?

   RESPONSE: Sorry for this confusion. When we examine the 39 disorders separately, we are focussing on each of these specific (narrow) disorders. When we focus on the 10 broad groups, we provide estimates for individuals who experience at least one of the disorders included in the broader group. We have now updated the methods section: "Individuals were considered to experience one of the broad categories if they were diagnosed with at least

one of the disorders included in the category" (page 10, lines 284-286).

8. Discussion page 12 top: you mention dementia here but it is not one of the 39 chosen conditions that you concentrate on in this paper (…and see comment above: did you take the different spots in ICD where dementia is coded?)

   RESPONSE: None of the 39 selected conditions showed MRRs that increased over time after onset. However, we think that this is still an important result to discuss. Consequently, we have changed the sentence to "Finally, MRRs did not increase over time after onset for any of the selected 39 conditions; however, MRRs increased over time for few disorders (e.g. dementia; ICD-10 code F00) when looking at all 1,803 disorders" (page 13, lines 387-389). Dementia was included as different specific 3-level ICD-10 codes.

9. Discussion page 12: what future study are you planning to further explore a potential impact of air pollution?

   RESPONSE: Several co-authors of this study are members of the BERTHA project (BigData Centre for Environment and Health) and the NordicWelfAir project (Interdisciplinary Nordic project funded by Nordforsk), which aim at using individual-level data to assess environmental exposures in Denmark (BERTHA) and the Nordic countries (NordicWelfAir). Air pollution is a prominent environmental threat, and as part of BERTHA and NordicWelfAir, several studies have been carried out in the past few years (e.g. Antonsen et al 2020, Horsdal et al 2019, Thygesen et al 2020, Raaschou-Nielsen et al 2020, Khan et al 2019, Holst et al 2020, Mok et al 2021). In one of these studies (Raaschou-Nielsen et al 2020), air pollution was found to be associated with mortality, and we wanted to perform sensitivity analyses adjusting the estimates of our study for air pollution. We believe this is useful to emphasize the possibility of this type of linkage using Danish registers, but more comprehensive studies focusing on specific health conditions and examining the effect of more pollutants are necessary to better understand the role of air pollution as a potential confounder between such health conditions and later mortality.

10. Discussion page 12: what do you mean with 'late-onset conditions'? For dementia that would read as onset at older ages but for cancer, that may be the case for some types of cancer but certainly not generalisable

    RESPONSE: We have left only dementia as example of late-onset disorder (page 15, line 447).

11. Discussion page 12: the single sentence about COVID-19 seems a little gratuitous: either expand on the topic or leave it out.

    RESPONSE: We have deleted the sentence (page 14, lines 407-408).

12. Limitations: you do not mention one important limitation: a finding of excess mortality risk or reduced life span associated with a particular diagnosis may not be related to the disease per se but reflecting common underlying risks even if there is no evidence of a direct relationship. Many diseases are linked with upstream risks like poverty or poor education. These in turn cause many other more proximal risks to be more common. If the outcome of interest has a link to poverty it takes on the baggage of all other risks that are elevated with poverty leading to excess deaths that are not 'due to' the condition of interest but confounded by the excess baggage of risk factors this person carries. In other words, you may not be estimating a true counterfactual: the absence of a disease of interest may not take away the fact that someone is

poor or has low education and therefore lots of other things predisposing to premature mortality.

RESPONSE: This is a very important point, and we completely agree with the reviewer. Thanks for prompting us to include extra text on this issue. We are very respectful of the 'complex web of causation' and we do not pretend that our analyses capture precise cause-and-effect relationships. Our study is a descriptive study in which we provide a range of epidemiological and mortality metrics for a comprehensive list of health conditions. We hope that our study can stimulate more focused epidemiological research questions that are better suited to single-disorder focus studies. Even then, causality is difficult to infer.

We have now included the following paragraph in the discussion: "It is important to note that a range of factors can influence mortality in those with specific health conditions. We do not propose a causal relationship between the health conditions and subsequent mortality. Observed associations could be explained by underlying factors that are associated both with morbidity and mortality (e.g. socio-economic or environmental factors). Additionally, the onset of a health condition can have impacts on lifestyle, daily habits and socio-economic characteristics, which in turn might mediate the association with subsequent mortality" (page 13, lines 392-397). Additionally, we have included one sentence in the abstract: added "The association between the different disorders and mortality could be confounded by underlying risk factors associated both with the disorder and mortality" (page 4, lines 104-105).

13. Limitations: you mention that you only capture diagnoses from hospital encounters and that you may therefore be missing conditions for which people largely seek care with GPs or do not seek care. A consequence for those conditions is that you may be selecting more severe cases of the disease who are more likely to appear in your disease registry and hence overestimate their mortality risk.

    RESPONSE: This is an important limitation, acknowledged in the discussion section. We have now expanded this paragraph to make it clearer: "The study does not include patients with disorders that more likely were treated by the general practitioner or who were not treated at all. While this limitation might have little impact on disorders such as cancer, psychotic disorders, or renal failure, the true prevalence and incidence of disorders like allergy, mild depression, or alcohol dependence might be underestimated, and their associated excess mortality overestimated <u>since the mortality metrics are based on the subset of individuals with more severe diseases that are seen and treated in secondary care</u>" (page 15, lines 432-434).

14. Appendix: by choosing the whole F chapter as 'mental disorders' you partially include dementia but not cases coded to G30 and G31. It will depend on local coding practices which codes are preferred. There are more examples of disease that straddle different ICD chapters and certainly those that fall across multiple smaller ICD groupings you created.

    RESPONSE: We decided to use the entire F chapter as mental disorders because it is described in the ICD-10 as 'Mental, behavioural and developmental disorders' and because it has been used in previous studies (e.g. Pedersen et al. 2014, Plana-Ripoll et al. 2019, Momen et al. 2020). However, we have provided the results for each specific ICD-10 code in the data visualization site and all the statistical code to perform the analysis, which would allow other researchers to apply for the same (or other) data and replicate the analyses for specific

combinations of ICD-10 codes of interest.

15. Appendix: your list of ICD categories uses mainly the S and T chapters with 'nature of injury' codes but you add 'cause of injury' codes for suicide and violence. There would be overlap between those: e.g. someone getting assaulted (X59-Y09) with as a consequence a head injury (S00-S09): that would be one individual and one injury episode.
    RESPONSE: We have included the ICD-codes used by the Danish Health Authority to report all hospital contacts. There might be many cases of individuals receiving two or more diagnoses (even on the same day). These individuals will contribute information to the estimates for each of their diagnoses. We have particular interest in how different diagnoses cluster within individuals across time (i.e. comorbidity) and we hope to explore the impact of comorbidity on these health metrics in future studies.

Reviewer #2:

This study tried to estimate a wide range of health metrics in Denmark from 2000 to 2018 using a comprehensive population-based cohort including all residents of Denmark. Although some estimates produced in this study are useful for policy making and population health research, I don't think there is enough innovation and unique contribution for a original research paper. Most of the health metrics (the important ones) estimated in this study have already been estimated by IHME using a more comprehensive and rigorous approach. I believe that GBD studies also utilized this national cohort for their Danish estimates. I doubt this study make much additional contribution. In addition, I found one major assumption about remission made in this study particulary contraversial, which makes the results less reliable or useful.

RESPONSE: Thank you for this feedback. We believe that this paper is innovative and contributes for original research, and we try to discuss the general issues provided in this general comment.

First, with respect to GBD having access to these data and thus reducing the innovation of our study; alas, this is not correct. Several of the authors are formal collaborators on the GBD projects (including participating in Nordic-specific GBD papers). The information on burden of disease is published in annual aggregated formats, which in turn, are included in the GBD estimates, but the estimates used in the GBD are not based on Danish registers. Danish registers (including those on hospitalizations and mortality) are only available to the health authorities and researchers based in Denmark. The GBD estimates rely on publications and reports (see complete list of data sources for Denmark from GBD's Global Health Data Exchange website https://ghdx.healthdata.org/gbd-2019/data-input-sources?components=9&locations=78); thus, our publication can contribute to improving the estimates for many of the diseases the GBD include in their annual estimates. Additionally, other researchers would also benefit from our study, given that the estimates could be included in systematic reviews or meta-analyses. Without comprehensive papers like this one, these data would only be available if researchers based in Denmark were interested in exploring a specific disease, decided to do so, and published the results with as many details as we are providing.

Second, it is true that we are reporting some estimates that are also reported by the GBD for a particular year (e.g. 2019), but our estimates are based on highly detailed individual-level data over a longer period of time. We were able to use information on every time someone received a diagnosis and the exact date of death. Additionally, we are also

providing important new health metrics that are not reported by the GBD. Years of Life Lost (YLL) provided by the GBD focuses on potential life lost after death (remaining life expectancy at time of death is estimated). In this study, we focus on remaining life expectancy after diagnosis. Consequently, disorders that are rarely included as causes of death are not considered as fatal disorders in the GBD study, but we can estimate the reduction in life expectancy associated with these disorders. For example, major depressive disorder has zero Years of Life Lost in the GBD Study (because it cannot be considered as cause of death according to their assumptions), but here we observed that individuals with a diagnosis of major depressive disorder have an average life expectancy 8 years shorter than the general population of same sex and age. This information is extremely relevant and is not currently provided by the GBD.

With respect to remission, we would have liked to include information on remission to the study. Unfortunately, this information is not available through hospital registers. The reviewer may not be aware that remission rates in GBD are estimated mostly via DISMOD2 and applied as a 'top down' process. Thus, it is impossible for GBD to link person-level hospital data with risk of mortality – this can be done with high quality registers. While the estimates of life expectancy must be interpreted with caution, all other estimates (incidence, age-at-onset, age-at-death, and mortality rates) are not affected by the lack of remission information (please see response to comment #6 below).

Here are my specific comments

1. Please provide line numbers for easier reference

   RESPONSE: We apologise for that and have now provided line numbers running continuously.

2. Data used in this study are not publicly available and some restrictions may apply

   RESPONSE: We have made publicly available all aggregated data and analysis code. However, we are not allowed to share individual-level data due to the current laws. Researchers interested in access to the underlying data can apply to the Danish Health Data Authority (www.sundhedsdatastyrelsen.dk).

3. Page 5, Introduction: "There is a need to better understand the impact on life expectancy of non-fatal disorders…" GBD studies also produced years of lives lost due to disability (YLDs) and thus DALYs, which can reflect burden due to the 'upstream' causes or risk factors. Actually, when calculating the YLDs, the incidence/proportion of different stages of a condition (four stages of cancers) and their corresponding disability weights were all carefully estimated to produce proper YLDs for each condition. So, I don't think this paragraph is well grounded.

   RESPONSE: We are aware that YLDs are useful to quantify the burden of disease, and we have published several papers based on Danish registers estimating YLDs for mental disorders (e.g. Weye et al 2021). However, YLDs account for only the non-fatal burden (i.e. the health loss experienced with living with this condition), not the implications of the conditions to ***premature mortality****, which is the focus of our current study.*

4. Page 5, Introduction: "…have assumed a fixed age-of-onset of 15 years…" So, if age-of-onset is 15 years, does it mean the age of onset is 15 years old? It's a bit unclear. It's also really hard to believe that age of onset for mental disorders is 15 years old.

   RESPONSE: We agree with the reviewer – it is hard to believe that the age of onset for everyone with a mental disorder is exactly 15 years old. Unfortunately, in the past, because of lack of better methods, previous studies have estimated life expectancy at time of

diagnosis assumed a fixed age of onset (e.g. 15 years for mental disorders, 20 years for type 1 diabetes, 55 years for colon cancer). We agree with the reviewer that this is not an optimal way to estimate life expectancy, which has led to the development of more valid methods. The 'life years lost' method we are applying in this study overcomes this limitation, by considering the real (or observed) age at diagnosis. We have included 'old' to make it clearer that we are talking about the age of onset. "For example, studies estimating life expectancy have assumed a fixed age-of-onset of 15 years <u>old</u> for those with mental disorders [4,5], 20 years <u>old</u> for those with type 1 diabetes [6], and 55 years <u>old</u> for those with colon cancer [7]" (page 6, lines 162-163).

5. Page 6, Introduction: "limited range of mortality-related estimates (e.g. the GBD only presents YLLs)." That's not true. GBD studies actually produced a wide range of health metrics, including YLLs, YLDs, DALYs, all-cause and cause-specific mortality rates, etc.

   RESPONSE: Sorry, we have deleted this sentence (page 7, line 180). In fact, GBD presents both YLLs and mortality rates (our mistake).

6. Page 7 and page 13: The authors ignored remission/recovery period for all diseases (one of their assumptions). This assumption does not make much sense for most infectious diseases that does not last long, do not affect health once fully recovered and can re-occur to the same person multiple times.

   *RESPONSE: Information on remission or recovery is not available through hospital registers. Fortunately, most of the estimates that we provide (e.g. incidence, age-at-onset, age-at-death, mortality rates, etc.) **are not affected by the lack of remission information**. However, the estimates on life expectancy are based on mortality rates from disease onset and onwards. Consequently, they can only be interpreted for individuals experiencing the mortality rates of the diseased from onset and onwards, which is equivalent to assuming that the disorders are chronic. This is a standard limitation in all register-based studies and all estimates based on life expectancy, but we made the assumption as an explicit limitation in our study. The 39 selected health conditions presented in the manuscript are based on an algorithm to identify chronic conditions using registers; thus, we believe the estimates of life expectancy are valid for these conditions. On the website (that shows the estimates for all 1,803 disorders), we have provided the following sentence next to each estimate related to life expectancy: "The estimate of life expectancy is based on the assumption that the diagnosed will experience the mortality rates of the diagnosed during the entire life (after diagnosis), which might be plausible for chronic disorders but not for acute ones". The user can then evaluate if the specific condition could be considered as chronic or not. Additionally, we have provided a sentence in the discussion to make it clearer: "This study did not include information on remission or other comorbid disorders; the group of individuals with a specific disorder can therefore be interpreted as persons who have had a diagnosis of the disorder regardless of whether they have other disorders or whether they have recovered afterwards. <u>Estimates of life expectancy are based on mortality rates from onset and onwards; thus, they can be interpreted mostly for chronic conditions</u>" (page 15, lines 437-438).*

7. The authors found that air pollution has little effects on the mortality rates. However, since Denmark does not have severe air pollution issue, I don't think this finding is true for many developing countries where air pollution is moderate or poor.

   RESPONSE: We agree with the reviewer that our study is based on estimates from Denmark and might not generalize to other countries, and have updated a sentence in the discussion

section: "While our estimates may be reflective of high-income countries, it remains unknown the extent to which our findings generalize to other countries with different healthcare systems or levels of air pollution" (page 15, lines 443-445).

Reviewer #3:

1. This is a useful, innovative way to examine disease burden in high-income countries. The MRRs are reasonably clear in methodology and presentation. However, the calculations for LYLs and the use of the method published earlier is not clear, and leaves the reader hanging. Further details of the calculations and a worked example in the appendix would be helpful. An appendix table showing the difference for say 39 conditions using the LYL used here versus a fixed LYL approach would be helpful- how does the new method alter priority setting?

   RESPONSE: Thank you for your positive comment and helpful feedback. Sorry about the explanation of the 'life years lost' method. We have now included a worked example in the appendix with additional explanations (Figure 1 in S1 Text). The method assuming a fixed age of onset is outdated, and we did not want to clutter our paper or data visualization site with estimates that are not accurate. Previous studies have discussed the utility of the 'life years lost' versus the past methods not accounting for age of onset or methods based on age at death (Andersen 2017, Plana-Ripoll et al 2020, Weye et al 2020). We have included a sentence in the discussion: "Additionally, it allows for the evaluation of the impact of particular disorders on premature mortality, regardless of how the individual died. A recent study using this method based on Danish registers [13] has shown that all mental and substance use disorders are associated with a reduction in life expectancy, although only few of them are generally included as cause of death" (page 14, lines 415-418).

2. As well, the main uncertainty here will not be sampling, which leads to the narrow CIs for most conditions, but two factors: (i) Misclassifications of the causes of death particularly at older ages, where COD data are less certain- so some exploration of the Danish death registry data for ill-defined contributions to COD by age would be helpful, and if possible stratifying the analyses into causes with low misclassification and those with high may be a useful appendix; (2) As mentioned, but not detailed in any sensitivity analyses, the variation in age at onset for conditions.

   RESPONSE: These are good points. Regarding the misclassification of causes of death, we agree with the reviewer and have acknowledged it in the discussion section "Since date of death is considered to be accurate, all-cause mortality is not affected by potential misclassification. However, there could be some misclassification of the specific cause of death, given that only 5% of deaths in Denmark are examined by autopsy [30]. However, given that all deaths were classified into two broad categories (natural and external causes), misclassification is less likely" (page 14, lines 439-443).

   Causes of death are prone to misclassification (the so-called 'garbage codes'). Fortunately, we were able to use data that has already been "cleaned" after the national quality adjustments by the National Board of Health. One of our co-authors has recently published a study investigating the quality of causes of death data in Denmark (Mikkelsen et al. 2020). Fortunately, only 8.1% of causes of death were insufficiently specified, and some of them represented general conditions (e.g. neoplasms of uncertain or unknown behaviour). Thus, we are confident that the classification into natural and external causes of death will be accurate.

In relation to the variation in age at onset for the conditions, we have incorporated the observed age at onset for all conditions. Since we believe this is important, we have mentioned in the discussion the impact of different ages of onset into mortality estimates (page 13, lines 371-379). Additionally, for all health conditions, we have reported age-at-onset distribution, age-specific incidence rates, age-specific mortality rates (and mortality rates ratios), and age-specific remaining life expectancy.

3. Minor point that the discussion results for COPD versus vascular should be in the results, and not presented in the discussion.
   RESPONSE: We have moved these sentences to the results section (page 11, lines 317-322).

4. Finally, Figure 5 is way too complicated , it should either be simplified- say to the top 20 leading causes of death, or leading one in each ICD10 chapter.
   RESPONSE: Sorry about this figure, but we hope that the interested reader can use our interactive data visualization site in order to drill down into the many data points (e.g. on the website, the reader can hover over a point for more details). In addition, it is informative to have the three panels next to each other (counts of disorders, MRRs, and LYLs). We have added the following text to the figure legend: "We encourage the reader to explore the estimates in more detail at http://nbepi.com/atlas."

REFERENCES

Andersen PK. Life years lost among patients with a given disease. Stat Med. 2017;36: 3573–3582.

Antonsen S, Mok PLH, Webb RT, et al. Exposure to air pollution during childhood and risk of developing schizophrenia: a national cohort study. The Lancet Planetary health. 2020;4(2):e64-e73.

Holst GJ, Pedersen CB, Thygesen M, et al. Air pollution and family related determinants of asthma onset and persistent wheezing in children: nationwide case-control study. Bmj. 2020;370:m2791.

Horsdal HT, Agerbo E, McGrath JJ, et al. Association of Childhood Exposure to Nitrogen Dioxide and Polygenic Risk Score for Schizophrenia With the Risk of Developing Schizophrenia. JAMA network open. 2019;2(11):e1914401.

Khan A, Plana-Ripoll O, Antonsen S, et al. Environmental pollution is associated with increased risk of psychiatric disorders in the US and Denmark. PLoS Biol. 2019;17(8):e3000353.

Mikkelsen L, Iburg KM, Adair T, et al. Assessing the quality of cause of death data in six high-income countries: Australia, Canada, Denmark, Germany, Japan and Switzerland. International Journal of Public Health 2020; 65: 17-28.

Mok PLH, Antonsen S, Agerbo E, et al. Exposure to ambient air pollution during childhood and subsequent risk of self-harm: A national cohort study. Prev Med. 2021;152(Pt 1):106502.

Momen NC, Plana-Ripoll O, Agerbo E, et al. Association between Mental Disorders and Subsequent Medical Conditions. New England Journal of Medicine. 2020;382: 1721–1731.

Raaschou-Nielsen O, Thorsteinson E, Antonsen S, et al. Long-term exposure to air pollution and mortality in the Danish population a nationwide study. EClinicalMedicine. 2020;28:100605.

Pedersen CB, Mors O, Bertelsen A, et al. A comprehensive nationwide study of the incidence rate and lifetime risk for treated mental disorders. JAMA Psychiatry. 2014;71: 573–581.

Plana-Ripoll O, Pedersen CB, Holtz Y, et al. Exploring Comorbidity Within Mental Disorders among a Danish National Population. JAMA Psychiatry. 2019;76: 259–270.

Plana-Ripoll O, Canudas-Romo V, Weye N, et al. lillies: an R package for the estimation of excess Life Years Lost among patients with a given disease or condition. Plos One. 2020;15: e0228073.

Thygesen M, Holst GJ, Hansen B, et al. Exposure to air pollution in early childhood and the association with Attention-Deficit Hyperactivity Disorder. Environmental research. 2020;183:108930.

Weye N, Momen NC, Christensen MK, et al. Association of Specific Mental Disorders With Premature Mortality in the Danish Population Using Alternative Measurement Methods. JAMA network open. 2020;3: e206646.

Weye N, Santomauro DF, Agerbo E, et al. Register-based metrics of years lived with disability associated with mental and substance use disorders: a register-based cohort study in Denmark. Lancet Psychiatry. 2021;8: 310–19.
