# Supplementary material for: Analysis of mortality metrics associated with a comprehensive range of disorders in Denmark, 2000 to 2018: A population-based cohort study
Source: PLoS Med. 2022 Jun 16;19(6):e1004023. doi: 10.1371/journal.pmed.1004023 (PMC9202944; doi:10.1371/journal.pmed.1004023)
Supplement: Attachment — Submitted filename: AtlasMortality letter - revision 2.docx [file pmed.1004023.s015.docx]

Dear Dr Moyer,

Many thanks for allowing us the opportunity to revise and resubmit this manuscript. Two versions of the revised manuscript have been uploaded (a ‘clean’ version, and one with track changes showing corrections). The page and line numbers refer to the version with track changes.

**Requests from Editors:**

1. Title: Please revise the title to: “Analysis of mortality metrics associated with a comprehensive range of disorders in Denmark, 2000-2018: A population-based cohort study” or similar. Please update the title in the text as well as the manuscript submission system.

*RESPONSE: We have changed the title as suggested.*

2. Abstract: Line 98: Please refer to an association between air pollution and MRRs, rather than an effect of air pollution.

*RESPONSE: We have now changed the sentence “Additionally, we examined the potential confounding effect of air pollution on MRRs” to “Additionally, we examined models that adjusted for measures of air pollution to explore potential changes on MRRs” (lines 98-99).*

3. Abstract: Methods and Findings: Line 107: Please make the sentence describing the limitations of the study more obvious. We suggest: “A limitation of the study is the possibility that the association between different disorders and mortality could be explained by underlying factors associated with both the disorder and mortality.” We suggest moving the statement about the adjustment for air pollution to the end of the results description.

*RESPONSE: We have moved the order of the sentence so that the statement about air pollution goes after all results and have reworded as suggested: “Adjustment for air pollution had very little impact on the estimates; however, a limitation of the study is the possibility that the association between the different disorders and mortality could be explained by other underlying factors associated with both the disorder and mortality” (lines 108-112)*

4. Author summary: Please re-format as bulleted points (3-4 per section) rather than in paragraph format. We suggest:

Why Was This Study Done?

-There have been many studies related to mortality linked to particular disorders, but these studies have not covered a comprehensive range of disorders.

-Previous studies have traditionally focused on relative measures of mortality (e.g. mortality rate ratios) or crude estimates of life expectancy that do not incorporate variation in the age of onset of the disorder.

-Here the researchers address these issues in a comprehensive atlas of mortality estimates based on Danish registers.

What Did the Researchers Do and Find?

-Based on 7,378,598 persons living in Denmark in 2000-2018, the researchers used national registers to identify individuals diagnosed with 1,803 specific categories of disorders.

-For each of these disorders, a panel of epidemiological and mortality metrics was estimated, including incidence rates, age-of-onset distributions, mortality rate ratios (MRRs) and life years lost (LYLs).

-Within a set of 39 selected medical conditions, mortality rates were larger and life expectancy shorter for 37 conditions compared to the Danish general population.

-The researchers have prepared an interactive data visualization to optimize the interrogation of their findings (<http://nbepi.com/atlas>).

What Do These Findings Mean?

-This study allows a more fine-grained analysis of the associations between a comprehensive set of disorders and mortality-related estimates.

-These findings can guide health research and serve as a benchmark to evaluate future health interventions.

*RESPONSE: We have now re-format this section using bullet points as suggested (lines 122-146)*

5. Introduction: Line 183-184: We suggest “Furthermore, differences between study designs complicates direct comparison of mortality between different disorders.” if accurate.

*RESPONSE: We have changed this sentence as suggested (lines 190-191).*

6. Methods: Analysis plan and Protocol: Line 195 and 276: Thank you for making the protocol and analysis plan publicly available. Please mention the included protocol and analysis plan in the supporting data files consistently throughout the text (e.g. S2_Text). Rather than including the web link in the text, we suggest including a reference for <https://doi.org/10.17605/OSF.IO/ZAFHU>.

*RESPONSE: We have included the link to OSF as a reference and cite it instead of including the webpage in the text (lines 202, 283, 294, 354 and all figure legends).*

7. Results: Line 345-347: Rather than including the web links for the data and interactive webpage in the text, we suggest including these in the reference list.

*RESPONSE: We have included the data and webpage as references (line 355).*

8. Results: Line 330-331: “Sex-specific estimates are available in supplementary S4 Figure.” If possible, please briefly summarize the key findings from the sex-specific analyses.

*RESPONSE: We have included a concise sentence about sex-specific analyses: “For most disorders, MRRs and LYLs were larger in males than in females, or similar for both sexes (sex-specific estimates are available in supplementary S4 Figure)” (lines 337-339).*

9. Author Contributions: Line 451: Please remove the “Authors’ Contributions” section from the main text and be sure all information is entered completely and accurately into the manuscript submission system.

*RESPONSE: We have deleted authors’ contributions from the text.*

10. References: Please check the formatting of each reference in the list. Please use the "Vancouver" style for reference formatting, and see our website for other reference guidelines <https://journals.plos.org/plosmedicine/s/submission-guidelines#loc-references>

*RESPONSE: We have now checked all references according to the guidelines.*

11. Journal name abbreviations should be those found in the National Center for Biotechnology Information (NCBI) databases. For example, please use “Lancet” as the journal title for references 1 and 3. Please use Br J Psychiatry as the journal title for reference 2. For reference 10, “PLoS One” should be the title. Please check the formatting throughout.

*RESPONSE: We have checked the formatting for all references.*

12. Figures 2, 3, 4, 5 and Figure S2: In the legends, please note the meaning of the red dashed line.

*RESPONSE: We have now written “The red line indicates equal mortality in the two groups (mortality rate ratio of 1; life years lost of 0)”.*

13. Table S2: Thank you for including the GATHER checklist. Please do not refer to the page numbers, and instead please replace these with paragraph numbers per section (e.g. "Methods, paragraph 1").

*RESPONSE: We have now used paragraph numbers per section.*

14. Figures S2, S3, S4: If possible, please also present these data in table format (similar to the presentation of Table S4).

*RESPONSE: We have now provided additional tables S5, S6 and S7 with the underlying data in figures S2, S3 and S4.*

**Comments from Reviewers:**

**Reviewer #1:**

I think the authors have done a good job at responding to comments.

*RESPONSE: Thank you for the positive comment.*

A few minor remaining issues:

Reviewer 1, comment 3: spelling out an acronym is not same as defining

*RESPONSE: The reviewer suggested to defined Life Years Lost in the abstract. We have now reworded to “For each of the disorders, a panel of epidemiological and mortality metrics was estimated, including incidence rates, age-of-onset distributions, mortality rate ratios (MRRs) and differences in life expectancy (estimated as life years lost; LYLs)” (line 97).*

Reviewer 1, comment 6: I'm largely OK with explanation but would expect this to be discussed in limitation section of discussion rather than hidden somewhere in appendix text.

*RESPONSE: We do not think that comparing life expectancy for those with a disorder to that in the general population (rather than those without the disorder) is a limitation, it is just a technicality of the life years lost metric. In fact, using this method with those without the disorder would be methodologically incorrect, as we mentioned in the appendix and in our previous revision.*

Reviewer 1, comment 8/14: you have not really addressed the issue of dementia codes that straddle two ICD chapters

*RESPONSE: We have included 1,803 different disorders in this study, and we decided to follow the ICD-10 classification. There are many health conditions that can be defined as a combination of several ICD-10 diagnoses, and it would be impossible for us to identify all of them. Addressing the specific nuances of the ICD-10 classification system is outside the scope of the current manuscript.*

Reviewer 2, comment about GBD: agree with most of the response. The statements on remission look a little odd; we have not used Dismod 2 for 15 years and I don't understand the reference to remission being a 'top-down process'

*RESPONSE: We apologise, we meant DisMod-MR version 2, not DisMod-2. By top-down process we meant that GBD does not have individual-level data, for example on who recovers from a particular condition. Instead, they have summary data that use to model recovery at the population level. This concept is not used in the manuscript.*

**Reviewer #2:**

I think the authors have adequately answered the reviewers' questions and addressed our concerns. I am satisfied with the authors' responses and happy to accept this revised version. Congratulations!

*RESPONSE: Thank you for the positive comment.*

**Reviewer #3:**

The authors have addressed most of my concerns. I quibble about the still too complicated Figure 5, but defer to the authors and editors to make this decision.

*RESPONSE: Thank you for the positive comment.*
